# Expression pattern of glycoside hydrolase genes in *Lutzomyia longipalpis* reveals key enzymes involved in larval digestion

**DOI:** 10.3389/fphys.2014.00276

**Published:** 2014-08-05

**Authors:** Caroline da Silva Moraes, Hector M. Diaz-Albiter, Maiara do Valle Faria, Maurício R. V. Sant'Anna, Rod J. Dillon, Fernando A. Genta

**Affiliations:** ^1^Laboratory of Insect Biochemistry and Physiology, Department of Biochemistry and Molecular Biology, Oswaldo Cruz InstituteFIOCRUZ, Rio de Janeiro, Brazil; ^2^Parasitology Department, Federal University of Minas GeraisBelo Horizonte, Brazil; ^3^Faculty of Health and Medicine, Division of Biomedical and Life Sciences, Lancaster UniversityLancaster, UK; ^4^National Institute of Science and Technology, Department of Molecular Entomology, Laboratory of Insect Biochemistry and PhysiologyRio de Janeiro, Brazil

**Keywords:** *Lutzomyia longipalpis*, digestion, β-1,3-glucanase, chitinase, lysozyme

## Abstract

The sand fly *Lutzomyia longipalpis* is the most important vector of American Visceral Leishmaniasis. Adults are phytophagous (males and females) or blood feeders (females only), and larvae feed on solid detritus. Digestion in sand fly larvae has scarcely been studied, but some glycosidase activities putatively involved in microorganism digestion were already described. Nevertheless, the molecular nature of these enzymes, as the corresponding genes and transcripts, were not explored yet. Catabolism of microbial carbohydrates in insects generally involves β-1,3-glucanases, chitinases, and digestive lysozymes. In this work, the transcripts of digestive β-1,3-glucanase and chitinases were identified in the *L. longipalpis* larvae throughout analysis of sequences and expression patterns of glycoside hydrolases families 16, 18, and 22. The activity of one i-type lysozyme was also registered. Interestingly, this lysozyme seems to play a role in immunity, rather than digestion. This is the first attempt to identify the molecular nature of sand fly larval digestive enzymes.

## Introduction

Phlebotomines are psychodid dipterans distributed over almost all faunal regions of the world and particularly abundant in temperate and inter-tropical regions. There are more than 600 described species to date. However, only 30 of these are epidemiologically important for pathogen transmission. Phlebotomine sand flies are able to transmit several diseases such as Leishmaniasis, which occur in approximately 98 countries and affects millions of people each year (WHO, 2014), as well as Bartonellosis (Tsai et al., 2011) and arbovirosis (Amaro et al., 2012).

Leishmaniasis are zoonosis caused by infection with *Leishmania* genus parasites and these are divided clinically in three forms (cutaneous, mucocutaneous, and visceral) depending on the parasite species. In the New World, Visceral Leishmaniasis is a severe systemic disease caused by *Leishmania* infantum (syn. *L. chagasi*) and transmitted by phlebotomines of the species *Lutzomyia longipalpis* (Romero and Boelaert, 2010; Harhay et al., 2011).

Like other Diptera, phlebotomines are holometabolous insects with adult and larval stages which exploit different food sources. Male and female adults feed on plant sap (or blood, in the case of females) (Brazil and Brazil, 2003; Soares and Turco, 2003) while larvae grow on decaying organic matter, mainly of plant origin, or animal feces. Wermelinger and Zanuncio (2001) found that *L. longipalpis* and *L. intermedia* larvae improved development until pupation when fed a variety of the diet described by Young et al. (1981), constituted by humus containing an intense proliferation of fungi. The authors suggested that this composition mimicked the larval substrate in the wilderness. However, despite these observations in the laboratory, little is known about the natural habitat of phlebotomine larvae (Feliciangeli, 2004).

Differences between larval and adult feeding habits of sand flies correlate with anatomical, physiological, and biochemical variations within the phlebotomine digestive tract. Therefore, it is not accurate to generalize about digestion throughout the development of these insects. Although some authors have shown the presence of both proteases and carbohydrases in sand flies (Dillon and El Kordy, 1997; Gontijo et al., 1998; Jacobson and Schlein, 2001; Ramalho-Ortigão and Traub-Csekö, 2003; Do Vale et al., 2007; Telleria et al., 2007, 2010; Sant'Anna et al., 2009), the role of digestible carbohydrases in phlebotomines and their potential participation in the digestion of microorganisms has not been systematically studied.

Moraes et al. (2012) described several glycosidases in larvae of *L. longipalpis* which may be related to the digestion of bacteria and fungi. This suggested that sand fly larvae have detritivorous feeding habits. The authors also assayed enzymatic activity of β-glucanases, chitinases, and lysozymes and confirmed that β-1,3-glucanases exhibited the highest specific activities in the luminal contents of the larval gut.

β-1,3-glucanases (βGlu) are glycoside hydrolases (GH) responsible for the hydrolysis of β-1,3-glucans which are found in the cell walls of fungi (Bartnicki-Garcia, 1968; Gorin and Spencer, 1968; Sietsma and Wessels, 1981) and phloem of higher plants as callose (Bacic et al., 1998). These enzymes are commonly found in the gut and salivary glands of insects such as *Spodoptera frugiperda* (Bragatto et al., 2010), *Tenebrio molitor* (Genta et al., 2009), and *Periplaneta americana* (Genta et al., 2003).

Besides β-1,3-glucanases, insects also express β-glucan recognition proteins (βGRPs), β-glucan binding proteins (GBP) and gram-negative bacteria binding proteins (GNBP) responsible for triggering the innate immune response through recognition of pathogen-associated molecular proteins (PAMPs) such as lipopolysaccharide (LPS), peptidoglycan, and β-1,3-glucan (Royet, 2004). These proteins are found mainly in hemolymph and fat body of insects (Ma and Kanost, 2000; Wang et al., 2005; Sun et al., 2011). Both β-1, 3-glucanases and β-glucan-binding proteins found in insects have been structurally assigned to family 16 of glycoside hydrolases (GH16) (Genta et al., 2009; Bragatto et al., 2010).

Chitinases (Chit) are enzymes able to hydrolyze chitin, a linear polymer of N-acetylglucosamine (GlcNAc) present in the cell wall of fungi as well as in insect structures such as cuticle, trachea, peritrophic matrix, and gut (Arakane and Muthukrishnan, 2010). All insect chitinases belong to family 18 of glycoside hydrolases (GH18) and are responsible for cuticle turnover, as well as digestion and degradation of the peritrophic matrix during molting (Zhang et al., 2011). GH18 are encoded by several groups of genes which are constituted by a multi-domain structural organization that includes: (1) 1–5 catalytic domains; (2) 0–7 cysteine-rich chitin-binding domains (CBD), and (3) serine/threonine-rich linker regions that can be heavily glycosylated (Merzendorfer and Zimoch, 2003; Arakane and Muthukrishnan, 2010).

Chitinases have been studied in different insect orders, in species such as *Anopheles gambiae* (Shen and Jacobs-Lorena, 1997; Zhang et al., 2011), *Aedes aegypti* (De la Vega et al., 1998), *Manduca sexta* (Kramer et al., 1993), *Bombyx mori* (Kim et al., 1998; Zhang et al., 2011), *Hyphantria cunea* (Kim et al., 1998), *Drosophila melanogaster* (Zhu et al., 2008), *Ostrinia nubilalis* (Khajuria et al., 2010), *Tribolium castaneum* (Zhu et al., 2008), *Tenebrio molitor* (Royer et al., 2002; Genta et al., 2006), and *Phlebotomus papatasi* (Ramalho-Ortigão et al., 2005; Coutinho-Abreu et al., 2010). Ramalho-Ortigão and Traub-Csekö (2003) isolated and characterized cDNA encoding a chitinase from the gut tissue of adult female *L. longipalpis* (Llchit1), which seems to be involved in the degradation of the peritrophic membranes and *Leishmania* migration toward the midgut epithelium during blood digestion.

Lysozymes (Lys) are glycoside hydrolases belonging to the glycoside hydrolases family 22 (GH22), whose function is to catalyze hydrolysis of glycosidic bonds between N-acetylmuramic acid (NAM) and N-acetylglucosamine (NAG). These molecules are constitutive components of the peptidoglycan layer of bacterial cell walls (Jollès and Jollès, 1984). Lysozymes are widely found in various organisms and are divided into six major groups. C-type lysozymes are probably the best studied and are found in several vertebrate and invertebrate taxa, including the vast class Insecta (Bachali et al., 2002; Harikrishnan et al., 2011). In addition to c-type lysozymes, other lysozymes have been studied, such as i-type lysozymes. This new class of lysozymes shows similarity with destabilases found in annelids (Zavalova et al., 2000), molluscs (Ito et al., 1999), and insects (Paskewitz et al., 2008). Zavalova et al. (2000) showed activity of i-type lysozyme in the cell wall of *Micrococcus lysodeikticus* and inhibition of such activity by anti-destabilase. However, Paskewitz et al. (2008) found no activity of i-type lysozyme in *Anopheles gambiae*.

An important hindrance for the study of sand fly enzymes is the diminute size of these animals, and their laborious and time-consuming maintenance of colonies, which hardly affords samples with amounts of protein enough for activity screenings. For this reason, the traditional strategies of enzyme purification, characterization, and sequencing, which were used in the study of other insect digestive glucanases and chitinases (Genta et al., 2009), are not applicable to this insect model. Anterior work from our group (Moraes et al., 2012) showed the presence of β-1,3-glucanase, chitinase, and lysozyme activities in the gut of sand fly larvae, but there are no description of any protein or DNA sequence related to digestive enzymes in these insects. In this work, we studied the expression pattern of different genes from families GH16, GH18, and GH22 in *L. longipalpis* and correlated two of them to the digestion of larvae. To our knowledge, this is the first report on the molecular identification of digestive enzymes in phlebotomine larvae.

## Materials and methods

### Sand fly rearing and larvae feeding

Insects used in all experiments were insectary-reared *L. longipalpis* from a colony originally started from individuals from Jacobina, Brazil, and maintained at the Laboratory of Insect Biochemistry and Physiology (Oswaldo Cruz Institute, FIOCRUZ). Adult sandflies were fed a 70% sucrose solution (w/v) *ad libitum*. Adult females were blood-fed with anesthetized hamsters (ketamine, 200 mg/kg) to trigger egg development. After oviposition, eggs were collected and reared to preserve the colony. All larval instars were fed a crushed mixture of rabbit feces, rabbit chow, and garden soil. Third and fourth instars were supplemented with a mixture of white soy protein (bran) and cereal flakes (Neston) (1:1).

### *in silico* mining of *L. longipalpis* ESTs library for glycoside hydrolases

To identify sequences of families 16 (β–1,3–Glucanases, GBP), 18 (chitinases), and 22 (lysozymes), glycoside hydrolases from different insect orders were identified and retrieved from CAZy (Carbohydrate-Active Enzyme database, http://www.cazy.org/). Subsequently, GH sequences obtained from CAZy were employed to perform a TBLASTX search (http://blast.ncbi.nlm.nih.gov/) to find similar sequences in a *Lu. longipalpis* EST library (Dillon et al., 2006) at the Sanger Institute website (http://www.genedb.org/Page/parasiteVectors).

### Analysis of GH sequences from the *L. longipalpis* EST library

Sequences retrieved from the *L. longipalpis* EST library were translated using the Translate tool (http://web.expasy.org/translate/) and compared against the non-redundant protein database from the National Center for Biotechnology Information (NCBI). Analysis of *Lu. longipalpis* ESTs similarity to GH families 16, 18, and 22 as well as prediction of ORF integrity was performed using the BLASTP tool. Translated sequences were further analyzed to identify signal peptides, O-type glycosylation, N-type glycosylation, and functional domains using PeptideIP Server 4.0 (Petersen et al., 2011; http://www.cbs.dtu.dk/services/SignalP/), NetOGlyc Server 3.1 (Julenius et al., 2005; http://www.cbs.dtu.dk/services/NetOGlyc/), NetNGlyc Server 1.0 (http://www.cbs.dtu.dk/services/NetNGlyc/), and database PFAM 26.0 (Punta et al., 2012; http://pfam.sanger.ac.uk/), respectively. Alignments were performed using ClustalW (http://ebi.ac.uk/Tools/msa/clustalw2/) (Hall, 1999).

### Phylogenetic analysis of *L. longipalpis* GH16, GH18, and GH22

Neighbor-joining phylogenetic trees for each gene family were constructed using insect protein sequences with PFAM domains GH16_beta_GRP (CD02179, β-1,3-glucanases and β-glucan binding proteins), Glyco_hydro_18 (PF00704, chitinases), and LYS (PF00062) and Destabilase (CD05497) for lysozymes. Trees were generated using MEGA5.05 (Tamura et al., 2011). Bootstrap values were set at 5000 replications.

### Dissections

4th instar larvae of *Lu. longipalpis* were rinsed in 200 μL of sterile 0.15 M NaCl solutions and anesthetized on ice. Larvae were dissected and the following structures removed: head (including foregut), midgut and hindgut (M+H) and carcass (rest of the body). Matching tissues were pooled in groups of 5 and then transferred to polypropylene vials containing 50 μL of TRI Reagent® (Sigma). Samples were flash-frozen and kept at −80°C until further RNA extraction.

### RNA extraction and cDNA synthesis

RNA was extracted from entire insects, head, M+H and carcass tissue samples, according to Diaz-Albiter et al. (2011). After extraction, total RNA was quantified using Nanodrop® (NanoDrop Technologies, Wilmington, USA). RNA was reverse-transcribed to cDNA using SuperScript III First-Strand Synthesis System (Invitrogen, San Diego, CA) following the manufacturer's protocol. cDNA was quantitated using Nanodrop and normalized to a concentration of 50 ng/μl.

### Tissue-specific expression of βGlu, GBP, Chit, and Lys

Polymerase Chain Reaction (PCR) and multiplex PCR were used to assess tissue-specific expression of β-Glu and GBP, Chit, and Lys in fourth instar larvae. For multiplex PCR, three specific primers were included in the same reaction, which allowed simultaneous amplification of more than one gene. Primer combinations were as follows: (1) 11b04, LamS2, 24g06, and Ribo60; (2) 96h07 and 154b12; and (3) 123b01 and 18f06. For sequences 88d12 and 14b06, PCRs were performed using only a pair of primers. All primers have listed in Table S1 in Supplementary Material. Amplification reactions were performed in a total volume of 20 μL containing 50 ng cDNA, 0.5 μM of each primer, 1×PCR reaction buffer, 0.2 mM each dNTP, 1.5 mM magnesium chloride and 0.025 U GoTaq® DNA polymerase (Promega). The parameters for PCR were: incubation at 94°C for 2 min followed by varying cycles of 94°C for 15 s, 55°C for 30 s, 72°C for 1 min and a final incubation (extension) of 72°C for 5 min. The number of cycles vary depending on the combination of primers used in the PCR and PCR-Multiplex reactions, as shown in Table S2 in the Supplementary Material. PCR products were analyzed by gel electrophoresis using agarose 2% (w/v), stained with ethidium bromide (0.5 μg/mL). Expression patterns were obtained by measuring the band intensity by densitometry using ImageJ software and then calculating relative expressions against a constitutive gene (AM088777, 60S ribosomal protein L3).

### Statistical analysis

All statistical analyses were performed using GraphPad Prism 5.0 for Windows, (San Diego California USA). Student's *t-*test for paired data was used. Comparison among and between groups was assessed using One-Way analysis of variance (ANOVA) with a *post-hoc* Tukey multiple comparison test. Results are expressed as the group mean ± s.e.m. Significance was considered when *p* < 0.05.

## Results

### In silico analysis of GHs sequences retrieved from the *L. longipalpis* EST library

A total of 206 sequences of glycoside hydrolases families 16, 18, and 22 from different insect species were found in CAZy, 85 of which belonged to family GH16, 47 to GH18, and 74 to GH22. They were employed to perform an *in silico* search targeting these three protein families in an *L. longipalpis* ESTs library (http://www.genedb.org/Page/parasiteVectors, Dillon et al., 2006). A total of 9 sequences were retrieved, 3 corresponding to clones of GH16 (identifiers: NSFM-140g04, NSFM-14b06, and NSFM-111b04), 5 corresponding to GH18 (identifiers: NSFM-18f06, NSFM-88d12, NSFM-24g06, NSFM-96h07, and NSFM-154b12) and 1 corresponding to GH22 (identifier: NSFM-123b01). Only five out of nine sequences displayed non-truncated open read frames (ORFs): NSFM-140g04 and NSFM-14b04 (GH16), NSFM-18f06 and NSFM-154b12 (GH18), and NSFM-123b01 (GH22). Moreover, prediction of signal peptides in these sequences suggests that their putative proteins are secreted. According to our analysis, hypothetical complete GH proteins have predicted molecular masses of 40–41, 43–47, and 15 kDa and estimated isoelectric points of 6.2–7.8, 6.8–8.4, and 4.9 (GHF16, GHF18, and GHF22, respectively).

Sequences of interest found in the *L. longipalpis* EST library were BLASTed against NCBI-NR and identified according to their best hit. All analyzed sequences were identified as proteins belonging to families GH16, GH18, and GH22, as shown in Table S3.

Amino acid sequences alignments of *L. longipalpis* proteins from GHF16, GHF18, and GHF22 with members from different insect groups exhibited highly-conserved regions (in Supplementary Material). *L. longipalpis* GH16 sequence of clone NSFM-140g04 was constituted by 385 amino acid sequence and highly-conserved regions when compared to other insect β-1,3-glucanases. These conserved regions also include typical family GH16 catalytic glutamate residues with proton donor-acceptor functions and are located at positions 196 and 201. Also, the sequence has a putative signal peptide with cleavage sites at positions 19 and 20 (Figure S1).

Putative β-glucan binding protein clones NSFM-111b01 and NSFM-14b06 found in the *L. longipalpis* EST library were constituted by 268 and 370 amino acid residues, respectively. These sequences shared similar regions with insect β-glucan binding proteins, also known as Gram-negative-binding proteins (GNBPs), (Figure S2). Since NSFM-111b01 was a truncated sequence at the 5′ region, it was not possible to evaluate the presence of a signal peptide. On the other hand, NSFM-14b06 did display a signal peptide with cleavage sites between positions 17 and 18. Interestingly, NSFM-111b01 and NSFM-14b06 lacked the catalytic glutamate residues responsible for hydrolysis of β-1,3-glucan otherwise present in β-1,3-glucanases (Figure S2).

Alignment of family GH18-like sequences showed that clones NSFM-154b12, NSFM-88d12, and NSFM-18f06 had a high level of conservation with other insect chitinases. Results of chitinases and chitinases-like domain organization analysis are summarized in Figure S3.

Clone NSFM-96h07 consisted of a small 113 amino acid region located in the protein catalytic domain. It was not possible to verify the presence of a signal peptide because 3′ and 5′ ORFs regions were truncated.

Insect chitinases share four different conserved regions (CR1 to CR4). Clone NSFM-154b12 consisted of 392 amino acids and showed a putative signal peptide with cleavage sites between amino acids 19 and 20. This sequence shared similarity in CR2 and CR3 (Figure S4). However, CR1 and CR4 were not found in this sequence. Clone NSFM-88d12 consisted of 296 amino acids and was truncated at region 5′, lacking a signal peptide and CR1. On the other hand, CR2, CR3, and CR4 were present and CR2 displayed the catalytic glutamate (E40) (Figure S5).

NSFM-24g06 consisted of 201 amino acid residues. The alignment of this sequence with other insect chitinases allowed identification of CR3 and also suggests that NSFM-24g06 is incomplete (Figure S6). The presence of CR1 and CR2 and the presence of catalytic residues could not be evaluated.

Clone NSFM-96h07 showed low conservation in amino acid sequence in CR1 and CR2 and no evidence of CR3 and CR4. Furthermore, NSFM-96h07 displayed a substitution of glutamate (E) with glutamine (Q75) at the hypothetical catalytic residue (Figure S7).

NSFM-18f06 consisted of 441 residues. This sequence showed a signal peptide with cleavage sites between amino acids 21 and 22. CR1 and CR2 were identified within this sequence. A serine residue (S152) was identified in CR2, unlike the all other sequence analyzed which had a glycine and alanine residue at that position. CR3 and CR4 were not identified in the alignment (Figure S8).

Lysozyme-like clone NSFM-123b01 consisted of 165 amino acid residues and appeared to be complete as suggested by the presence of a methionine residue and a stop codon. Furthermore, SignalIP showed the presence of signal peptide cleavage sites between amino acids 22 and 23, (Figure S9).

### Phylogenetic analysis of GH16, GH18, and GH22 sequences found in the *L. longipalpis* ESTs library

To classify all *L. longipapis* genes found in families GH16, GH18, and GH22 and to predict a physiological role of these putative enzymes, phylogenetic trees were constructed using a Neighbor-joining algorithm. Subgroups considered for phylogenetic analysis were β-glucanases and β-glucan binding proteins for family GH16, I to VIII-chitinase groups of families GH18 (Zhang et al., 2011), and i and c-type lysozymes for family GH22 (Bachali et al., 2002; Paskewitz et al., 2008). Analysis of GH16 was restricted to sequences from order Diptera while in GH18 and GH22 it considered several orders of insects.

**Figure 1:**
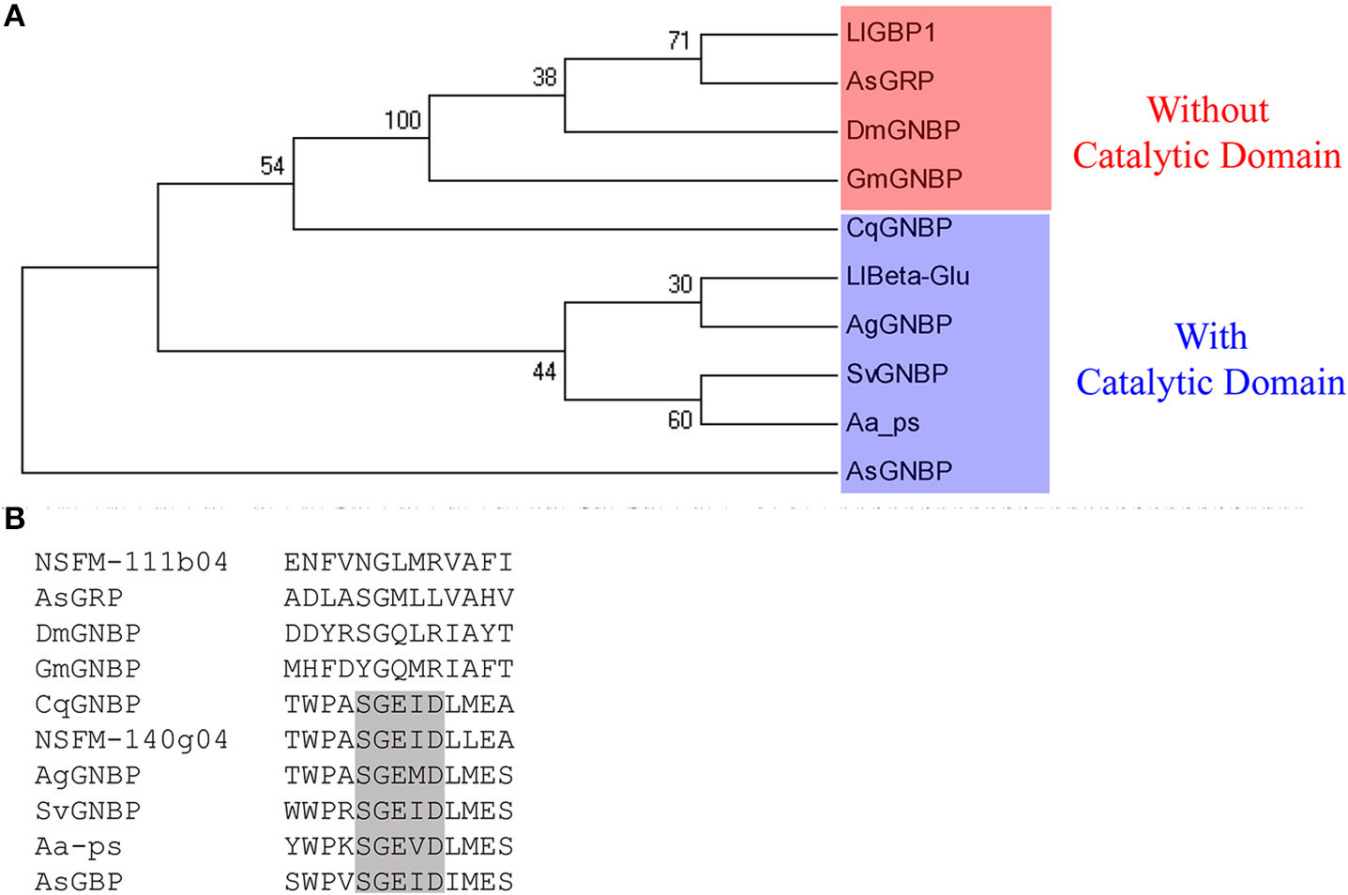
**Cladogram of selected GH16 proteins from the order Diptera. (A)** Phylogenetic tree was done using sequences from *Armigeres subalbatus* (accession number AAT99011), *Glossina morsitans morsitans* (ABC25063), *Drosophila melanogaster* (AAF33851), *Anopheles gambiae* (ACN38130), *Culex quinquefasciatus* (AEQ27734), and *Simulium vittatum* (ACH56895). *Actinophrys sol* (Porifera, BAG32349) was used as outgroup. The sequences NSFM-111b04 and NSFM-140g04 retrieved from *Lutzomyia longipalpis* EST are named as LlGBP1 and LlBeta-Glu, respectively. Bootstrap values were obtained by neighbor-joining method (software MEGA 5.05) using 5000 replications. **(B)** Aligment of the β-1,3-glucanases active site sequences used for phylogenetic analysis showing the presence of the catalytic region (black box).

Phylogenetic analysis of family GH16 revealed that sequences found in the *L. longipalpis* ESTs library separately groups with each of the two major clades found in this family: one clade includes sequences with high bootstrap, all lacking the catalytic glutamates (e.g., NSFM-111b04), and other clade which includes sequences with low bootstrap and catalytic residues (e.g., NSFM-140g04) (Figure 1A). Moreover, the latter clade forms a paraphyletic group with a *C. quinquefasciatus* sequence annotated in GenBank as a Gram-negative bacteria binding protein, which harbors the glutamate catalytic residues.

There was no evidence of any Pfam domain for NSFM-14b06. Furthermore, NSFM-111b01 and NSFM-14b06 sequences lacked the catalytic site responsible for hydrolysis of β-1,3-glucan otherwise present in β-1,3-glucanases (Figure 1B). Taking into account the presence and location of a catalytic site in the sequences within the clade of digestive enzymes, we named NSFM-140g04 as L1βGlu, whereas NSFM-111b04 and NSFM-14b06 were named LlGBP1 and LlGBP2, respectively.

Phylogenetic analysis of GH18 divided 4 *L. longipalpis* chitinases into different clades. To perform this, we extracted the GH18 chitinase catalytic conserved domains (Pfam 00704), except for the NSFM-96h07 sequence which did not contain such domain.

**Figure 2:**
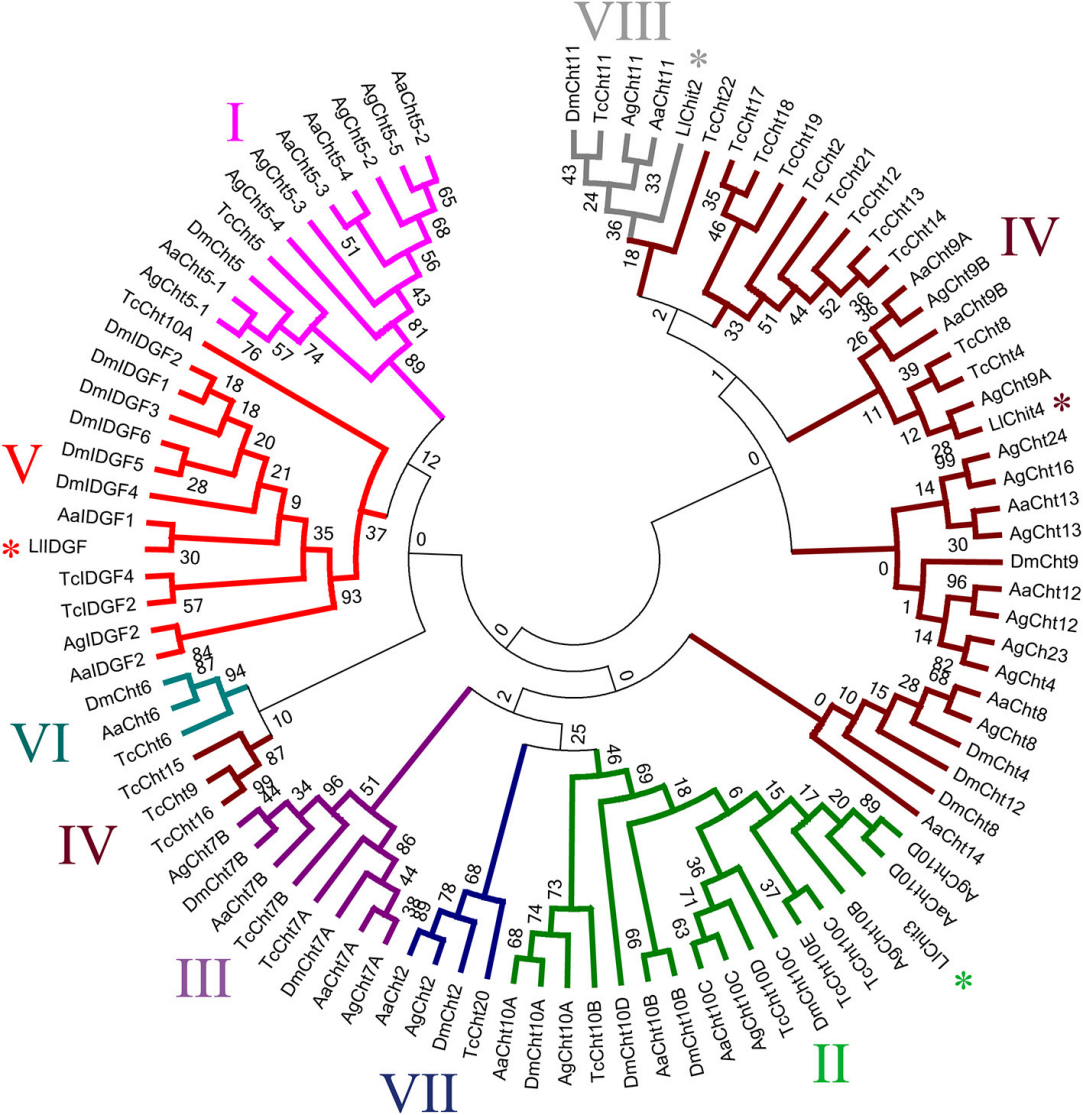
**Cladogram of selected insect GH18 protein sequences and positioning of *L. longipalpis* GH18 (LlChit2, LlChit3, LlChit4, and LlIDGF) in chitinase subgroups.** Sequences are from: *Aedes aegypti* (accession number XP_001657537; XP_001656234; XP_001656233; XP_001656232; XP_001656231; XP_001662588; XP_001650020; XP_001663097; XP_001656054; XP_001655973; XP_001654045; XP_001663568; XP_001655071; XP_001663099; XP_001660745; XP_001660748), *Anopheles gambiae* (XP_315650; XP_315351; HQ456129; HQ456130; HQ456131; HQ456132; HQ456133; XP_308858; XP_316448; XP_307732; XP_001238192; XP_310662; XP_316142; XP_314312; XP_319801; XP_001688641; XP_316256; XP_001237925), *Drosophila melanogaster* (NP_477298; NP_524962; NP_650314; NP_572598; NP_647768; NP_611542; NP_611543; EAA46011; NP_572361; NP_726022; NP_477258; NP_477257; NP_723967; NP_727374; NP_611321; NP_477081) and *Tribolium castaneum* (NP_001034516; NP_001073567; NP_001034524; XP_967813; NP_001036035; NP_001038094; NP_001038096; NP_001036067; XP_974461; XP_972802; NP_001036034; XP_973005; XP_973077; NP_001034515; XP_972719; XP_973161; XP_973119; XP_970191; NP_001034517; NP_001038095; NP_001038092; NP_001038091. Bootstrap values were obtained by neighbor-joining method (software MEGA 5.05) using 5000 replications. Branches marked with an asterisk correspond to *L. longipalpis* sequences.

In our analysis, chitinase subgroups I, II, III, V, VI, VII, and VIII formed monophyletic groups, while group IV formed a paraphyletic group (Figure 2). According to clade division, *L. longipalpis* sequences were divided as follows: NSFM-154b12 into type VIII, NSFM-88d12 into type II, NSFM-24g06 into type IV, and NSFM-18f06 into type V (Figure 2). After the chitinases classification from our filogenetics analysis, putative chitinases were named as follows: NSFM-154b12 is LlChit2, NSFM-88d12 is LlChit3, NSFM-24g06 is LlChit4, NSFM- 96h07 is LlChit5, and NSFM-18f06 is LlIDGF. The name LlChit1 was not used in this work to avoid confusion with the sequence described by a Ramalho-Ortigão et al. (2005; LlChi1).

**Figure 3:**
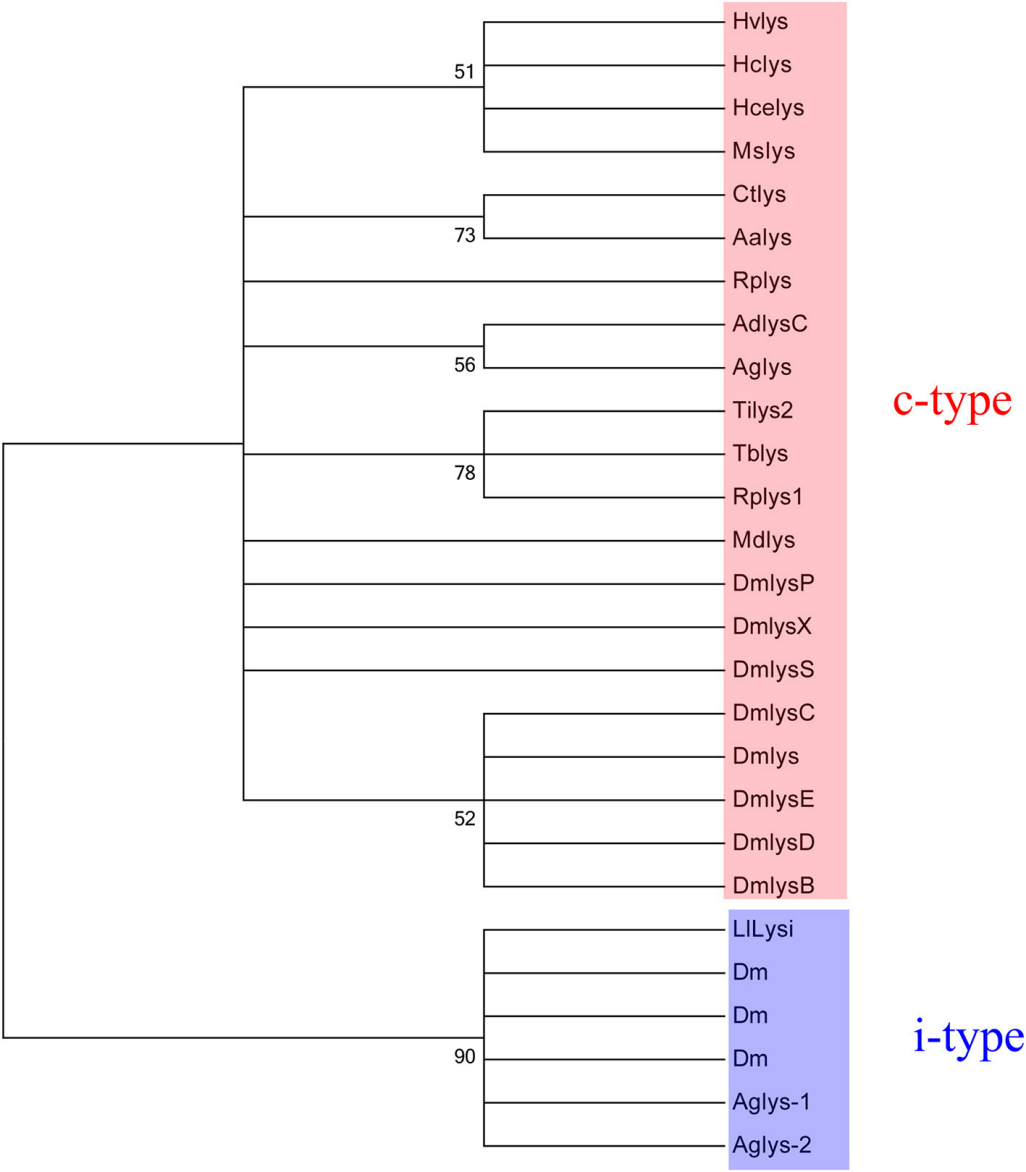
**Cladogram of selected insect GH22 protein sequences (c and i-type lysozymes) and LlLysi.** Sequences are from: *Anopheles darlingi* (accession number AAB61345), *Anopheles gambiae* (AAC47326; AY659931; EF492429), *Drosophila melanogaster* (AAF47445; AAF47453; CAA21317; AAF57939; AAF57940; AAF47452; AAF47451; AAF47450; AAF47449; AAF47448; CAA80225), *Heliothis virescens* (AAD00078), *Hyalophora cecropia* (AAA29189), *Hyphantria cunea* (AAA84747), *Manduca sexta* (AAB31190), *Musca domestica* (PC4062), *Triatoma infestans* (ABI94387), *Triatoma brasiliensis* (AAU04569), *Rhodnius prolixus* (ABX11554; ABX11553), *Culex tarsalis* (ACJ64375), *Aedes aegypti* (CAC19819). Bootstrap values were obtained by neighbor-joining method (software MEGA 5.05) using 5000 replications.

The phylogenetic tree of lysozymes from insects was divided in two clades: c-type lysozyme and i-type lysozymes, forming monophyletics groups in each clade (Figure 3). Positioning of the NSFM-123b01 sequence showed that this sequence belonging to the i-type lysozyme family (Figure 3), named in this paper as LlLysi.

### Expression of β-1,3-glucanases, chitinases, and lysozyme during development of *L. longipalpis*

**Figure 4:**
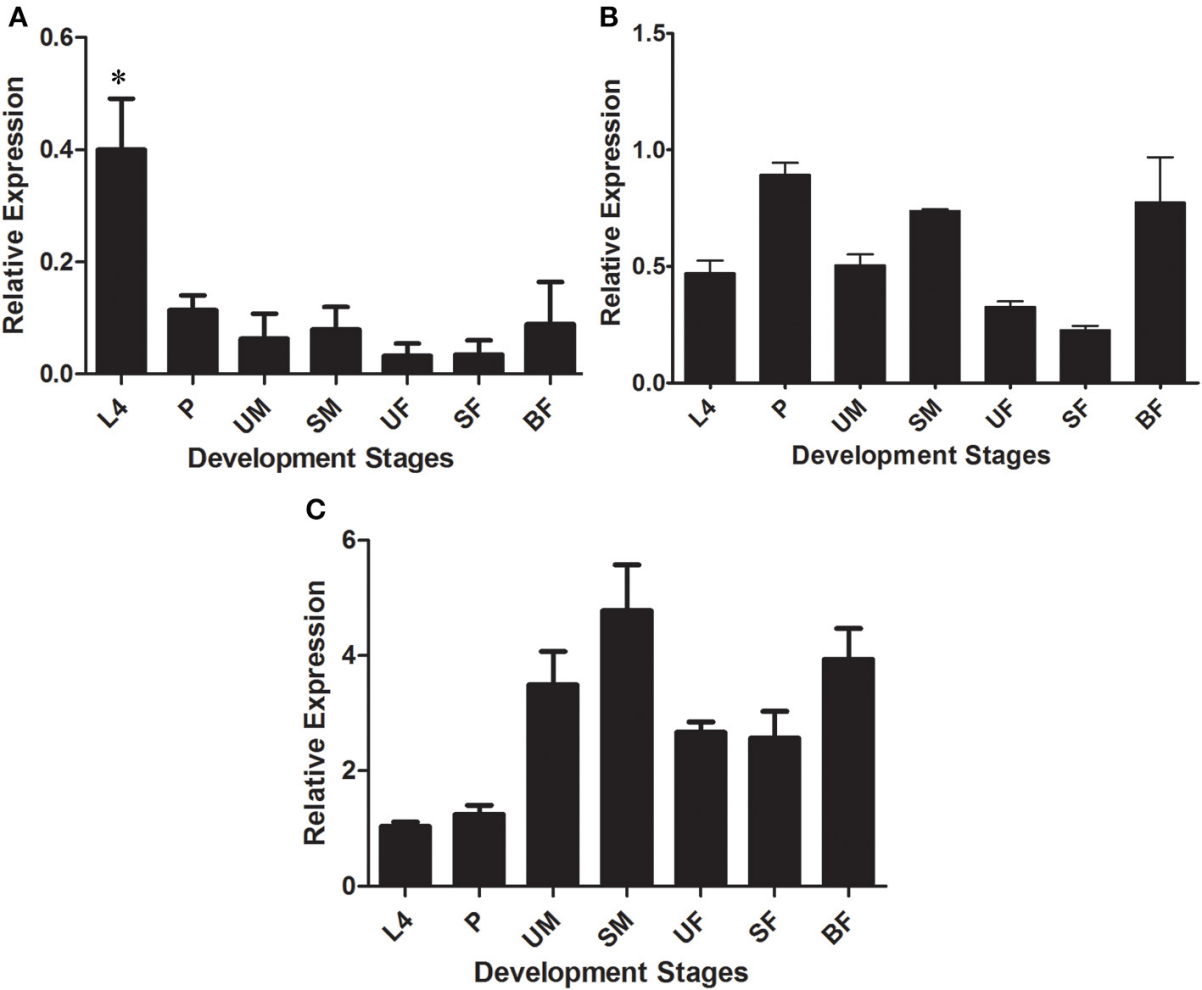
**Relative expression of β-1,3-glucanases (LlβGlu) (A) and β-glucan binding proteins (LlGBP1 and LlGBP2) (B,C, respectively) in different stages of development of *Lutzomyia longipalpis* as determined by Multiplex RT-PCR (Ribosomal protein 60 used as constitutive gene).** The experimental groups used in the experiments were larvae (L4), pupae (P), unfed male adults (UM), sugar-fed male adults (SM), unfed female adults (UF), sugar-fed female adults (SF), and blood-fed female adults (BF). Bars represent the mean ± SE from 3 independent experiments. The statistics were done using ANOVA with Turkey post-test and student's *t*-test. Asterisks indicate statistically significant differences in the samples.

The expression of putative glycoside hydrolase-coding transcripts from families 16, 18, and 22 (β-glucanases/β-glucan binding proteins, chitinases, and lysozyme, respectively) was evaluated at different development stages and dietary conditions of *L. longipalpis*, namely larvae (L4), pupae (P), unfed male adults (UM), sugar-fed male adults (SM), unfed female adults (UF), sugar-fed female adults (SF), and blood-fed female adults (BF). Among GH16 sequences, LlβGlu showed a significantly higher relative expression in the L4 larval stages (0.40 ± 0.09) when compared to the other stages of development (*p* < 0.05) (Figure 4A). In contrast, binding proteins were similarly expressed throughout all stages (LlGBP1, Figure 4B) or poorly expressed in the larval stage (LlGBP2, Figure 4C).

LlGBP1 showed a higher relative expression in P (0.89 ± 0.05), SM (0.74 ± 0.01), and BF (0.7 ± 0.1). Relative expression was significantly higher in P compared to L4 (*p* < 0.005), and UF and SF (*p* < 0.001). Furthermore, the relative expression of SM was significantly different compared with UF and SF (*p* < 0.0001). Expression in blood-fed females was statistically difference compared to SF (*p* < 0.05) (Figure 4B).

LlGBP2 showed higher relative expressions in the adult stage, specially in UM (3.4 ± 0.5), SM (4.7 ± 0.7), and BF (3.9 ± 0.5). However, expression in UM was only significantly different from L4 (*p* < 0.05), SM expression values were significantly different to L4 (*p* < 0.01) and P (*p* < 0.05), while values in BF were different from L4 (*p* < 0.01) and P (*p* < 0.01) (Figure 4C).

**Figure 5:**
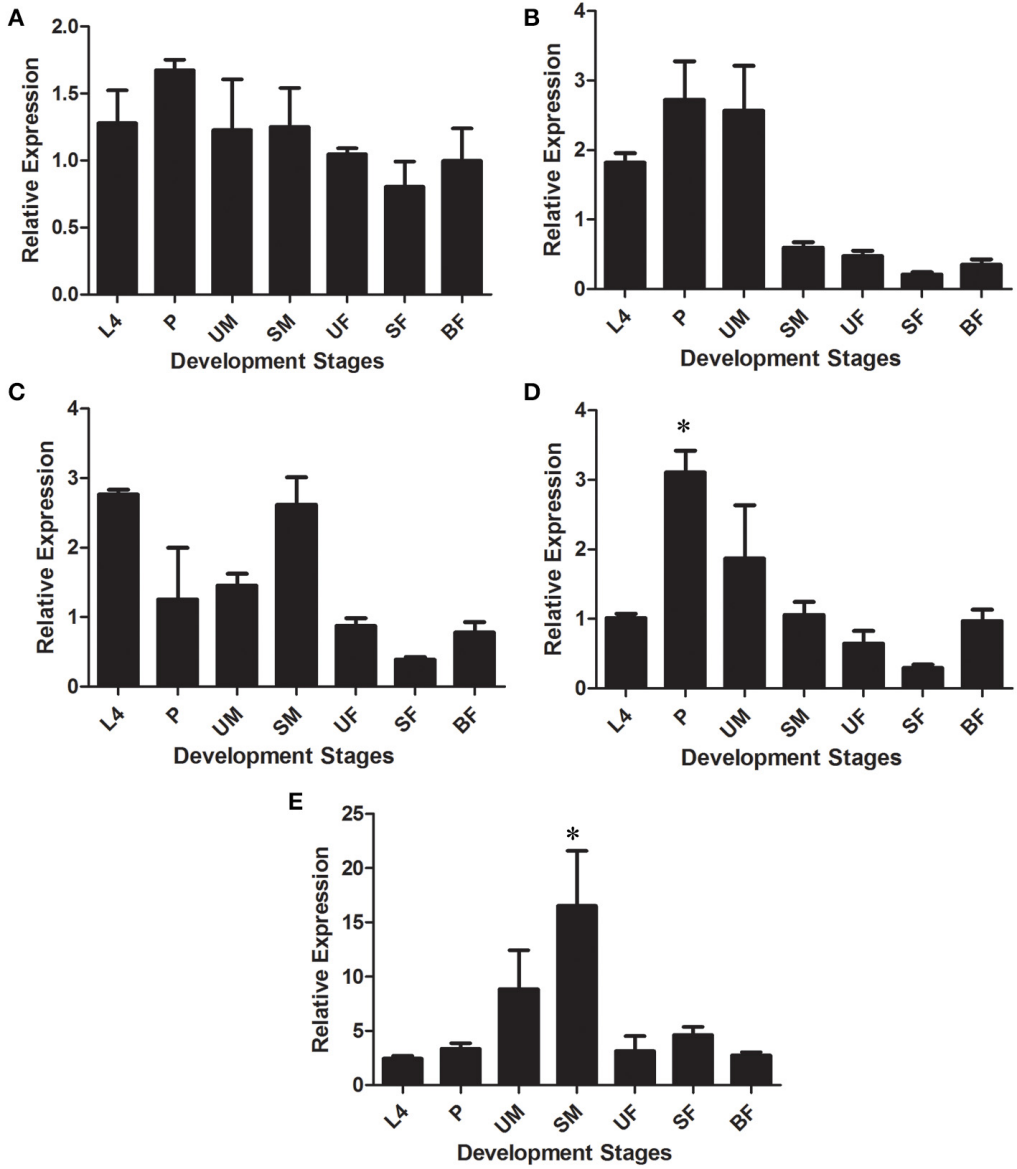
**Relative expression of GHF18 members in different stages of development of *Lutzomyia longipalpis* as determined by Multiplex RT-PCR (Ribosomal protein 60 used as constitutive gene).** Chitinases amplified were: **(A)** LlChit2; **(B)** LlChit3; **(C)** LlChit4; **(D)** LlChit5, and **(E)** LlIDFG. The experimental groups used in the experiments were larvae (L4), pupae (P), unfed male adults (UM), sugar-fed male adults (SM), unfed female adults (UF), sugar-fed female adults (SF), and blood-fed female adults (BF). Bars represent the mean ± SE from 3 independent experiments. The statistics were done using ANOVA with Turkey post-test and student's *t*-test. Asterisks indicate statistically significant differences in the samples.

Chitinases-like transcripts also showed different expression patterns throughout the sand fly developmental stages. LlChit2 showed no significant differences between experimental groups (Figure 5A).

LlChit3 showed higher relative expression in L4 (1.8 ± 0.1), P (2.7 ± 0.6) and UM (2.6 ± 0.6). Values were significantly different when compared to SM (*p* < 0.01), while values in P were different from SM and UF, SF, and BF (*p* < 0.05) (Figure 5B). LlChit4 showed high relative expression levels in L4 (2.8 ± 0.1) and SM (2.6 ± 0.4). Expression levels in L4 were significantly different from UF, SF, and BF (*p* < 0.001). The sugar-fed males showed significant differences compared to all adult female groups UF, SF, and BF (*p* < 0.05) (Figure 5C).

LlChit5 showed higher relative expression in P (3.1 ± 0.3) compared to all groups (*p* < 0.05) except UM (Figure 5D). LlIDGF showed significant differences in relative expression in SM (16 ± 9) compared to groups L4, P, UF, SF, and BF (*p* < 0.05) (Figure 5E).

**Figure 6:**
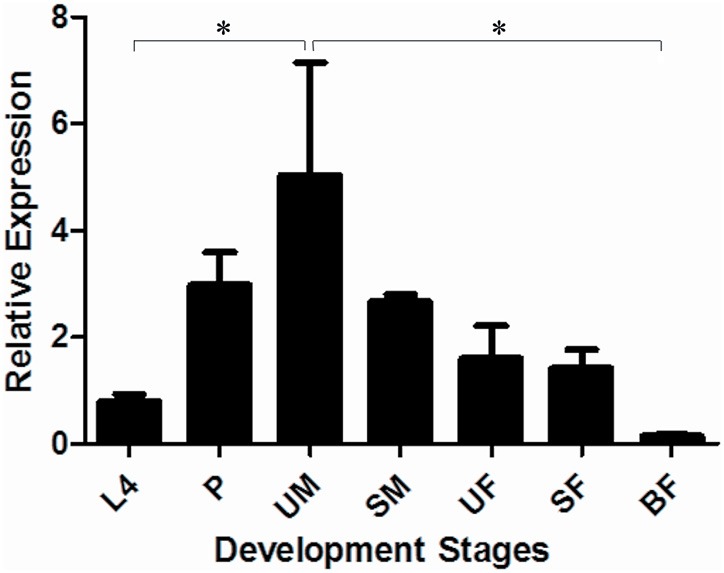
**Relative expression of lysozyme (LlLysi) in different stages of development of *Lutzomyia longipalpis* as determined by Multiplex RT-PCR (Ribosomal protein 60 used as constitutive gene).** The experimental groups used in the experiments were larvae (L4), pupae (P), unfed male adults (UM), sugar-fed male adults (SM), unfed female adults (UF), sugar-fed female adults (SF), and blood-fed female adults (BF). Bars represent the mean ± SE from 3 independent experiments. The statistics were done using ANOVA with Turkey post-test and student's *t*-test. Asterisks indicate statistically significant differences in the samples.

LlLysi showed higher relative expression values UM (5 ± 2). However, significant differences were only observed in L4 and BF (*p* < 0.05), as shown in Figure 6.

### Tissue-specific expression of β-1,3-glucanases, chitinases, and lysozyme in larvae of *L. longipalpis*

**Figure 7:**
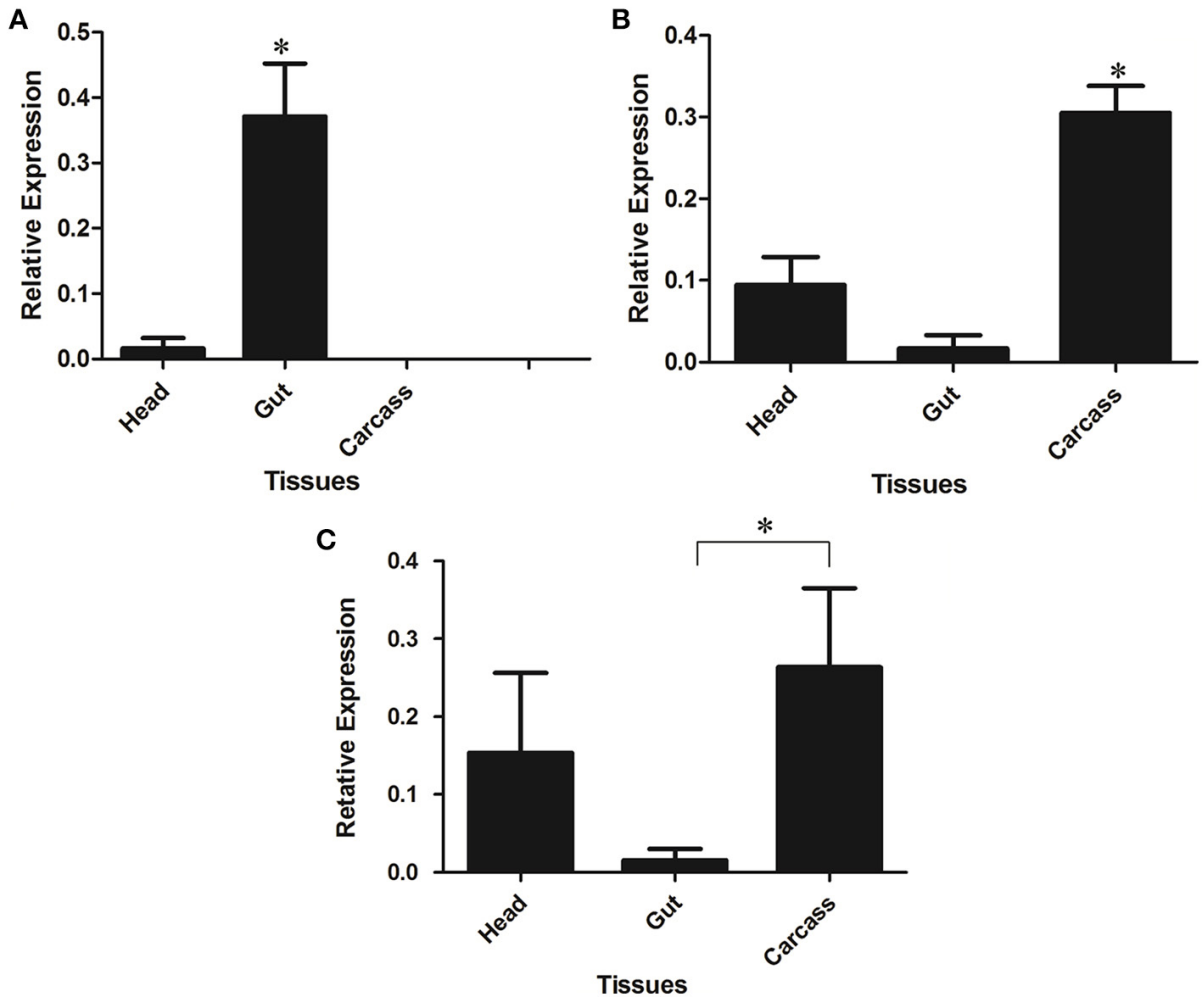
**Relative expression of GHF16 (A) β-1,3-glucanase (LlβGlu) and (B) β-glucan binding proteins LlGBP1 and (C) LlGBP2 in different tissues of *Lutzomyia longipalpis* L4 larvae as determined by Multiplex RT-PCR (Ribosomal protein 60 used as constitutive gene).** Bars represent the mean ± SE from 5 independent experiments. The statistics were done using ANOVA with Turkey post-test and student's *t*-test. Asterisks indicate statistically significant differences in the samples.

LlβGlu showed a significantly higher relative expression level in larval guts when compared to other tissues and it appears to be gut-specific (*p* < 0.01, Figure 7A). On the other hand, expression of LlGBP1 was significantly higher in carcass (Figure 7B) and LlGBP2 showed higher expression levels in head and carcass when compared to the gut (*p* < 0.01) (Figure 7C).

**Figure 8:**
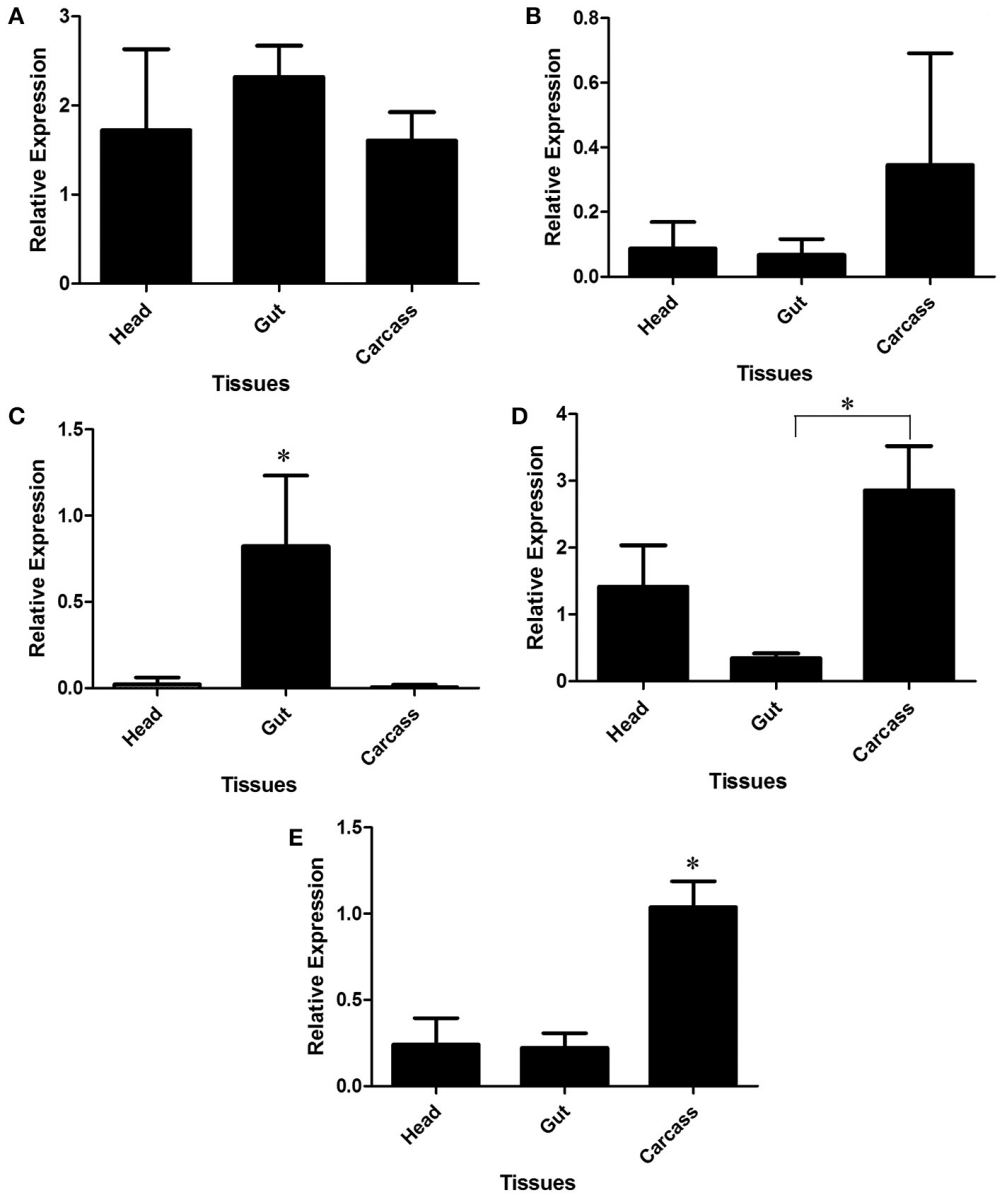
**Relative expression of GHF18 chitinases and chitinase-like proteins in different tissues of *Lutzomyia longipalpis* L4 larvae as determined by Multiplex RT-PCR (Ribosomal protein 60 used as constitutive gene).** Chitinases amplified were: **(A)** LlChit2; **(B)** LlChit3; **(C)** LlChit4; **(D)** LlChit5 and **(E)** LlIDGF. Bars represent the mean ± SE from 5 independent experiments. The statistics were done using ANOVA with Turkey post-test and student's *t*-test. Asterisks indicate statistically significant differences in the samples.

LlChit2 and LlChit3 showed no statistically significant differences between any tissue-specific samples (Figures 8A,B, respectively). However, data suggest a slightly higher expression in carcass samples for LlChit3 (Figure 8B).

**Figure 9:**
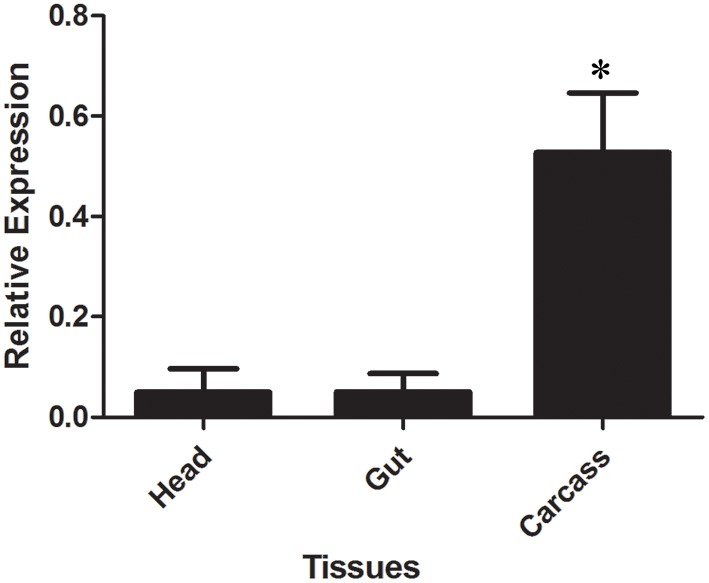
**Relative expression of i-type lysozyme (LlLysi) in different tissues of *Lutzomyia longipalpis* L4 larvae as determined by Multiplex RT-PCR (Ribosomal protein 60 used as constitutive gene).** Bars represent the mean ± SE from 5 independent experiments. The statistics were done using ANOVA with Turkey post-test and student's *t*-test. Asterisks indicate statistically significant differences in the samples.

LlChit4 was more expressed in the gut tissue when compared to the head and carcasss of sand fly larvae (Figure 8C, *p* < 0.01). LlChit5 was more expressed in carcass when compared to gut samples (*p* < 0.01, Figure 8D). LlIDGF showed significantly higher expression values in carcass when compared to other tissues (*p* < 0.01, Figure 8E). LlLysi also showed higher expression levels in carcass compared to the head or intestine (*p* < 0.01, Figure 9).

## Discussion

### Digestion of microorganisms in detritivore insects and sand flies

Despite the wide variety of dietary sources used by insects in nature, feeding on decaying organic matter (i.e., plant debris and animal feces) is a recurrent evolutionary trait in several insect orders as Dictyoptera, Isoptera, Coleoptera, Diptera. Furthermore, some insects feed on organic matter decomposers such as fungi and are called fungivorous. These groups include some social insects from order Hymenoptera so specialized that they cultivate fungi to feed their own colonies (Chapela et al., 1994). Some species of beetles, such as *Dorcus rectus* feeds on decaying wood and its associated fungi in nature and can survive under laboratory conditions fed merely on fungi (Tanahashi et al., 2009).

*L. longipalpis* is a Dipteran from family Psychodidae and hematofagous females of this species are the main vector of visceral leishmaniasis in the New World. However, little is known about the feeding habits of their larvae in their natural environment. In experimental conditions, larval *L. longipalpis* and *L. intermedia* developed better when fed on a diet of fungi-rich humus which mimics larval substrate in the wilderness (Wermelinger and Zanuncio, 2001). Recently, breeding sites for sand fly larvae were described in the forest located at Amazonas State (Alencar et al., 2011) and in urban and peri urban areas in Southeast Brazil (Casanova et al., 2013). In the forest, sand fly larvae seem to be associated with the humus-rich soil near tree bases, and in urban and peri-urban areas the preferential breeding sites seem to be the soil at chicken sheds. Despite the limitations of these studies, it is quite relevant to perceive the strict association of sand fly larvae with microorganism-rich decaying organic matter in the wild.

Considering that, it is expected that sand fly larvae could exploit microorganisms as bacteria and fungi as a nutritional source. In this respect, their digestive enzymes must be capable of chemically disrupt the cell walls of these microorganisms, in order to release intracellular molecules as nucleic acids, proteins and storage sugars and lipids, which are essential to the insect development and metabolism. The main digestive enzymes related to fungal and bacterial cell disruption in insects are β-1,3-glucanase, chitinase, and lysozyme. Digesting β-1,3-glucanases from GHF16 were already described in detritivore insects from orders Dictyoptera (Genta et al., 2003), Coleoptera (Genta et al., 2009), Isoptera (Lucena et al., 2011). Digestive chitinases from GHF18 were described in detritivore Coleoptera (Genta et al., 2006), and digestive Lysozymes (GHF22) are a common feature of Dipteran Cyclorrapha (Terra and Ferreira, 2005). All these enzymes were found in a recent report on sand fly larval gut activities (Moraes et al., 2012) but, contrarily to the examples above, sand fly enzymes could not be molecularly identified by traditional biochemical techniques, due to the minimal size of these insects. In spite of that, our bioinfomatic and expression analysis of selected transcripts in *L. longipalpis* suggest that these larvae use GHF16 and GHF18 in their digestion, as observed in other insect orders.

### Functional specialization of GHF16 members in *L. longipalpis*

The *in silico* search for GH-like sequences in a *L. longipalpis* EST library showed the presence of three clones highly similar to family GH16 proteins (NSFM-14g04, NSFM-111b04, and NSFM-14b06). Previous sequence comparisons and phylogenetic analysis supported an evolutionary relationship between β-1,3-glucanases and β-glucan binding proteins from GHF16 (Pauchet et al., 2009; Bragatto et al., 2010; Hughes, 2012). The most evident features which distinguish these two functional groups are (1) the presence of catalytic glutamates in a conserved region which correspond to the active site in β-1,3-glucanases and (2) the presence of a conserved 100 amino acid N-terminal extension in β-glucan binding proteins. It has been proposed that the animal β-1,3-glucanase ancestral gene suffered a duplication before the differentiation of arthropods and molluscs (Bragatto et al., 2010) and, in this respect, insects should bear at least two copies of genes from GHF16.

Pauchet et al. (2009) also divided clades of Lepidopteran GHF16 sequences according to the absences or presences of catalytic residues. The authors suggested that such division happened through a duplication event of a gene in a common ancestor. This originated two major groups with different functions in insects: digestion and immune signaling. Recently, Hughes (2012) showed that GHF16 Pathogen Recognition Receptors (PRRs, which includes β-1,3-glucan binding proteins) and β-1,3-glucanase sequences are present in orders Coleoptera, Diptera and Lepidoptera, and suggested that this gene duplication event occurred before divergence of these holometabolous orders. The author also exhibited that only PRR-like sequences are found within Exopterigota (hemimetabolous), while only glucanase-like sequences are found in Isoptera. Such evidence supports the hypothesis that these two subfamilies underwent events of gene duplication before the origin of holometabolous insects (i.e., during the Carboniferous, approximately 300 million years ago).

Based on the criteria described above, NSFM-14g04 was classified as a β-1,3-glucanase (LlβGlu) while NSFM-111b04 and NSFM-14b06 were assigned to the β-glucan binding protein group (as LlBGP1 and LlBGP2). Alignment of these sequences with other insect GH16 showed that clone NSFM-14g04 contains the two glutamate residues which are important for catalysis. In contrast, NSFM-111b04 and NSFM-14b06 lack these residues, which suggests that these putative proteins do not have enzymatic activity. The presence of the N-terminal region typical for β-glucan binding proteins was not confirmed in LlGBP1, because this sequence is truncated at the N-terminal, but was confirmed in LlGBP2. LlβGlu, as expected, does not contain this extension, which was confirmed as LlβGlu appears as a complete coding sequence with a putative signal peptide. The presence of a putative signal peptide in LlβGlu and LlGBP2 sequences is coherent with the observations that some digestive insect β-1,3-glucanases follow the exocytic route for secretion (Bragatto et al., 2010) and that β-glucan binding proteins are soluble proteins secreted to the hemolymph, where they interact with members of the prophenoloxidase activating cascade (Lee et al., 2004).

The expression pattern of GHF16 members in *L. longipalpis* corroborated the classification based on sequence features. LlβGlu was more expressed in larvae and, at this stage, its expression is gut-specific. Gut-specific β-1,3-glucanases from GHF16 were already described in Lepidoptera (Pauchet et al., 2009; Bragatto et al., 2010), Coleoptera (Genta et al., 2009), and Isoptera (Bulmer et al., 2009). To our knowledge, this is the first description of this kind of protein sequences in Diptera. The role of insect gut β-1,3-glucanases is still controversial, as they were implicated in digestion of fungi and plant cell wall polysaccharides in some insects (Dictyoptera, Genta et al., 2003; Orthoptera, Genta et al., 2007, Coleoptera, Genta et al., 2009) but in recognition of gut-pathogens in others (Lepidoptera, Pauchet et al., 2009; Isoptera, Bulmer et al., 2009). Considering the detritivore habit of *L. longipalpis* larvae in the laboratories, with the ingestion of significant amounts of fungal tissue in the food (Moraes et al., 2012), we are tempted to suppose that the main role of LlβGlu is the disruption of ingested fungal cells, but more functional studies are necessary to confirm this hypothesis.

At the same time, LlGBP1 and LlGBP2 showed low levels of expression in the larvae and, at this stage, low levels of expression in the gut. In this respect, a role for these genes in larval digestion can be ruled out. Besides that, the expression patterns of LlGBP1 and LlBGP2 strongly suggest that they are involved in defense against pathogens, as they are expressed in tissues and stages more susceptible to infection. Both genes are highly expressed in males fed with sugar and blood fed females. The ingestion of bacterial pathogens by sand flies in sugar meals is a current topic of investigation (Telleria et al., 2013), as well as the multiplication of these micro organisms inside the blood meal in the sand fly gut (Diaz-Albiter et al., 2012). The fact that sand flies mount strong defenses in the gut during these physiological conditions suggests that they are fighting pathogens and, in fact, interfere with this phenomena result in activation of the prophenoloxidase cascade and insect death (Diaz-Albiter et al., 2011).

Nevertheless, there are subtle differences in the expression patterns between LlGBP1 and LlGBP2. LlGBP1 is highly expressed during the pupal stage, as well as LlGBP2 is highly expressed in unfed males. In this respect, these genes behave like their putative homologs in *Drosophila*, where GNBP1 (CG6895-PA), GNBP2 (CG4144), and GNBP3 (CG5008) are not expressed in the midgut at any stage, with higher expression levels in the embryo (GNBP2 and 3), pupae (all GNBPs) and adults (GNBP2 and 3), and preferential expression in the carcass or head (St. Pierre et al., 2014). It is possible that LlGBP1 is related to the prevention of infections during the pupal stage, which is extremely sensitive to infections. Besides that, it has been shown that unfed sand flies carry a significant amount of bacteria from the larval stage (Sant'Anna et al., 2014), which suggests that LlGBP2 could be involved in the specific protection against these recalcitrant microorganisms. The trans stadial passage of bacteria is a well-documented phenomenon in sand flies (Volf et al., 2002), and may have important implications in the development of strategies for the blocking of vectorial transmission of pathogens based in paratransgenesis. However, more functional studies are needed to confirm the roles of LlGBP1 and LlGBP2, especially after challenge with insect pathogens.

### Functional specialization of GHF18 members in *L. longipalpis*

Five sequences from GHF18 were retrieved from the *L. longipalpis* EST library (NSFM-96h07, NSFM-154b12, NSFM-88d12, NSFM-24g06, and NSFM-18f06). According to amino acid similarities, domain compositions, and phylogenetic analysis, insect proteins from GHF18 are classified into 8 groups, I-VIII (Zhang et al., 2011). A key feature in these proteins is the presence of a catalytic glutamate in the sequence DWEYP at the consensus region 2 (CR2), which is used as a marker for enzymatic activity. GHF18 proteins without this residue are named chitinase-like proteins and considered devoid of hydrolytic activity. Besides that, functional studies were able to incriminate groups I and II of insect chitinases in chitin hydrolysis during molting, group III in the distension of wings and abdomen during morphogenetic development, and group IV as digestive enzymes. Group V is devoid of catalytic activity and is referred as Imaginal Disk Growth Factors, promoting cell proliferation in imaginal disks. Groups VI-VIII were described based only in sequence attributes, with no clear functional role assessed to this moment. In general, chitinases from all groups but IV are expressed in all stages of development, while the expression of group IV chitinases is restricted to specific stages. Many group IV chitinases are larval-specific, and gut-specific genes, being considered primarily as digestive enzymes. Some are involved in digestion or turnover of the peritrophic membrane (Ramalho-Ortigão and Traub-Csekö, 2003), but others, which lack the characteristic C-terminal Chitin Binding Domain, are believed to act in disruption of fungal cell walls without affecting the PM structure (Genta et al., 2006).

The domain organization of all *L. longipalpis* GHF18 sequences present in the ESTs database could not be assessed, because only two of them (NSFM-154b12 and NSFM-18f06) code for complete ORFs. These complete sequences contain a putative N-terminal signal sequence, which is a common feature in GHF18 insect chitinases (Zhang et al., 2011). This is coherent with chitinase putative secretion to the molting fluid, intestinal lumen, or to the extracellular matrix where it exerts its action on imaginal disk cells (Arakane and Muthukrishnan, 2010).

In spite of lacking complete N- or C-terminal ends, all GHF18 proteins from *L. longipalpis* were assigned to functional groups of insect chitinases II, IV, V, or VIII, being named LlChit2 (group VIII), 3 (group II), 4 and 5 (Group IV) and LlIDGF (group V). From these sequences, only LlChit2 and LlChit3 contain the conserved catalytic glutamate at CR2, being probably true chitinases. LlIDGF is a chitinase-like protein, and it was not possible to check for the presence of the catalytic glutamate in the case of LlChit4, because CR2 was missing in this sequence. In spite of that, LlChit4 is probably an active enzyme, as it groups with other true chitinases from group IV and contain the conserved PFAM domain Glyco_hydro_18 (PF00704). LlChit5 also aligned with proteins of group IV, but was not included in the phylogenetic analysis because the PFAM domain PF00704 was missing in its sequence. Because of that, the presence of the catalytic glutamate in region CR2 could not be really assessed, as its apparent substitution in LlChit5 sequence could be the result of the forced alignment of its truncated sequence. Another possibility is that LlChit5 use an alternative basic hydrolysis mechanism, based only in the nucleophilic catalysis from the acetamido group of the substrate (Hashimoto et al., 2000). More evidence is necessary to ascertain the catalytic nature of LlChit4 and LlChit5.

Chitinases from subgroups II, V, and VIII are typically expressed in all stages of insect development (Zhang et al., 2011). The expression pattern observed for LlChit2, 3, 5, and LlIDGF is consistent with this behavior. Indeed, LlChit2, 3, and 5 reach their highest expression levels in the pupal stage, and LlIDGF is more expressed in adult males. Besides that, in the larvae these genes are more expressed in the carcass (LlChit3, 5, and LlIDGF) or equally expressed in all larval tissues (LlChit2). These data strongly suggest that LlChit2, 3, 5, and LlIDGF are not involved in the digestion of sand fly larvae.

Interestingly, LlChit4, which is related to typical insect digestive chitinases from group IV, is more expressed in larvae and sugar fed males. Type IV chitinases contain a signal peptide, a single catalytic domain and mostly no CBDs (Genta et al., 2006). Additionally, LlChit4 is more expressed in the gut of larvae, which strongly suggest that this transcript correspond to the larval digestive chitinase of *L. longipalpis*. The higher expression in sugar fed males could be related to the proliferation of microorganisms in the gut of these insects, but this hypothesis still needs confirmation.

Similar results were observed by Khajuria et al. (2010), who demonstrated by phylogenetic analysis that a chitinase (OnCht) predominantly expressed in the gut of larval *Ostrinia nubilalis* was a type IV chitinase. A digestive chitinase was also found in the midgut of *T. molitor* larvae (Genta et al., 2006, TmChi).

In several hematophagous insects, digestive chitinases have an extremely important role in the degradation of type I peritrophic matrix (PM I). Zhang et al. (2011) showed expression of numerous chitinases in *Anopheles gambiae*, among them, a chitinase mainly expressed in the gut of adults (AgCht8).

Previous studies made by Ramalho-Ortigão and Traub-Csekö (2003) demonstrated the expression of chitinase (LlChi1) in the midgut of adult female *L. longipalpis*, whose expression seems to be induced after blood feeding. However, so far the expression of chitinases in larvae of *L. longipalpis* has not been demonstrated and studies to pursue a chitinase important for digestion of microorganisms present in the diet of the larvae are still scarce. The data obtained suggest that LlChit4 probably has that role.

To our knowledge, this is the first description of a larval gut chitinase sequence in sand flies. Larval gut chitinase activities were already described in *L. longipalpis* (Moraes et al., 2012) and *Aedes aegypti* (Souza-Neto et al., 2003), these enzymes being probably involved in the digestion of chitin rich structures as the fungal cell wall. Gut-specific expression of chitinase genes in Diptera was already described in *Anopheles gambiae* larvae (Zhang et al., 2011), where group IV chitinases AgCht8 and AgCht13 are majorly expressed only in the midgut at this stage. It is interesting to notice that in some cases dipteran larval digestive chitinases seem to be coded by genes different from their adult counterparts, a pattern already observed for other gut genes, as trypsins and peritrophins (Venancio et al., 2009).

### Analysis of the GHF22 sequence found in adult ESTs library of *Lutzomyia longipalpis*

Lysozymes are present in a wide variety of organisms (from viruses to plants and animals) and can be classified based on sequence similarity into 5 groups: type c, type g, type i, plant, and bacterial lysozymes (Bachali et al., 2002). Insect lysozymes are commonly members of family GH22, being involved in the arthropod innate immune response against bacteria but also in the digestion of food microorganisms in detritivores (Jollès and Jollès, 1984; Hultmark, 1996). The most well characterized insect digestive lysozyme is the gut activity from *Musca domestica* larvae. This enzyme is a c-type lysozyme (Cançado et al., 2007) with specializations in its structure which are related to its optimum pH at the midgut strong acidic conditions (Cançado et al., 2010), which is a typical feature of Diptera Cyclorrapha (Terra and Ferreira, 1994). As the midgut luminal pH of sand fly larvae is strongly alkaline (Do Vale et al., 2007) it could be possible that their digestion of bacteria rely on a different type of lysozyme.

In recent years studies have revealed the existence in insects of a second family of lysozymes, type i, for which at least 20 examples have been identified in the phylum Mollusca, Nematoda, Annelida, and Arthropoda (Ito et al., 1999; Zavalova et al., 2000; Bachali et al., 2002; Paskewitz et al., 2008).

Therefore, considering the possible role of lysozyme in the degradation of dietary microorganisms in sand flies, a search was also made for GHF22 members in the EST database of *L. longipalpis*. Our analysis revealed only one transcript similar to insect lysozymes, called LlLysi (NSFM-123b01), whose sequence was complete including a putative N-terminal signal peptide. Moreover, comparison of this sequence in the Pfam database showed that LlLysi contains a destabilase domain.

Similarity to Destabilase is common among i-type lysozymes. The leech destabilase has such a name from their ability to break or destabilize fibrin blood clots in the ingested blood (Zavalova et al., 2000). Using this relationship, the leech destabilase was used as a pattern to pursue i-lysozymes in the genome of *Anopheles gambiae* (Paskewitz et al., 2008).

Aiming to classify the lysozyme found in the EST library of *L. longipalpis*, we performed a phylogenetic analysis using the neighbor-joining algorithm. In this analysis it was possible to demonstrate the separation of c- or i-lysozymes in two monophyletic groups. LlLysi was classified in this analysis as i- lysozyme. To our knowledge, this is the first description of this type of lysozyme in sand flies.

LlLysi was expressed throughout all developmental stages of *L. longipalpis*, expression being the highest in unfed males. This could be related to the presence of high amounts of bacteria in recently emerged adults of *L. longipalpis* (Sant'Anna et al., 2014). Besides that, a very low level of LlLysi expression was found in L4 larvae, being concentrated in the carcass, excluding the possibility of LlLysi being a larval digestive enzyme. Similar studies were done in the mosquito *A. gambiae*, which produces two i-type lysozymes, called Lys i-1 and i-2. Lys i-1 was expressed in all stages of mosquito development, being more expressed in the ovaries of females, followed by Malpighian tubules and fat body. Lys i-2 was more abundant in the fat body of adults (Paskewitz et al., 2008).

Thus, in sand flies, the lysozyme gene involved in the larval digestion of bacteria, which corresponds to the activity described by Moraes et al. (2012) is different from the i-lysozyme transcripts represented in adult EST databases. Our studies raise the interesting possibility that sand fly larval gut lysozyme could be in fact more related to the c-lysozymes from other Diptera, but more studies are necessary aiming the molecular identification of this enzyme.

## Conclusion

The comparative analysis of sequences present in EST databases as well as the determination of expression patterns during development and the tissue location of transcripts from families GH16 and GH18, allowed us to identify the genes related to the β-1,3-glucanase and chitinase which are involved in digestion of microorganisms in larvae of *L. longipalpis*. However, the sequence of lysozyme present in adult EST databases (LlLys, family GH22) is apparently a type i Lysozyme involved in immunity, having no relationship to larval digestion of bacteria. To our knowledge, this is the first molecular identification of digestive enzymes in the larvae of sand flies, which are important vectors of several diseases, including Leishmaniasis. These findings may have a profound impact on the understanding of the biology of these disease vectors, as well as for the development of new strategies for vector control.

## Author contributions

Conception and design of the work: Rod J. Dillon, Maurício R. V. Sant'Anna, Fernando A. Genta. Obtainment of experimental data: Caroline da Silva Moraes, Hector M. Diaz-Albiter, Maiara do Valle Faria, Maurício R. V. Sant'Anna, Fernando A. Genta. Data analysis: Caroline da Silva Moraes, Maiara do Valle Faria, Hector M. Diaz-Albiter. Writing and revision of the manuscript: Caroline da Silva Moraes, Hector M. Diaz-Albiter, Rod J. Dillon, Fernando A. Genta.

### Conflict of interest statement

The authors declare that the research was conducted in the absence of any commercial or financial relationships that could be construed as a potential conflict of interest.
