# Supplementary material for: Expression pattern of glycoside hydrolase genes in Lutzomyia longipalpis reveals key enzymes involved in larval digestion
Source: Front Physiol. 2014 Aug 5;5:276. doi: 10.3389/fphys.2014.00276 (PMC4122206; doi:10.3389/fphys.2014.00276)
Supplement: Supplementary file 1 [file DataSheet1.PDF]

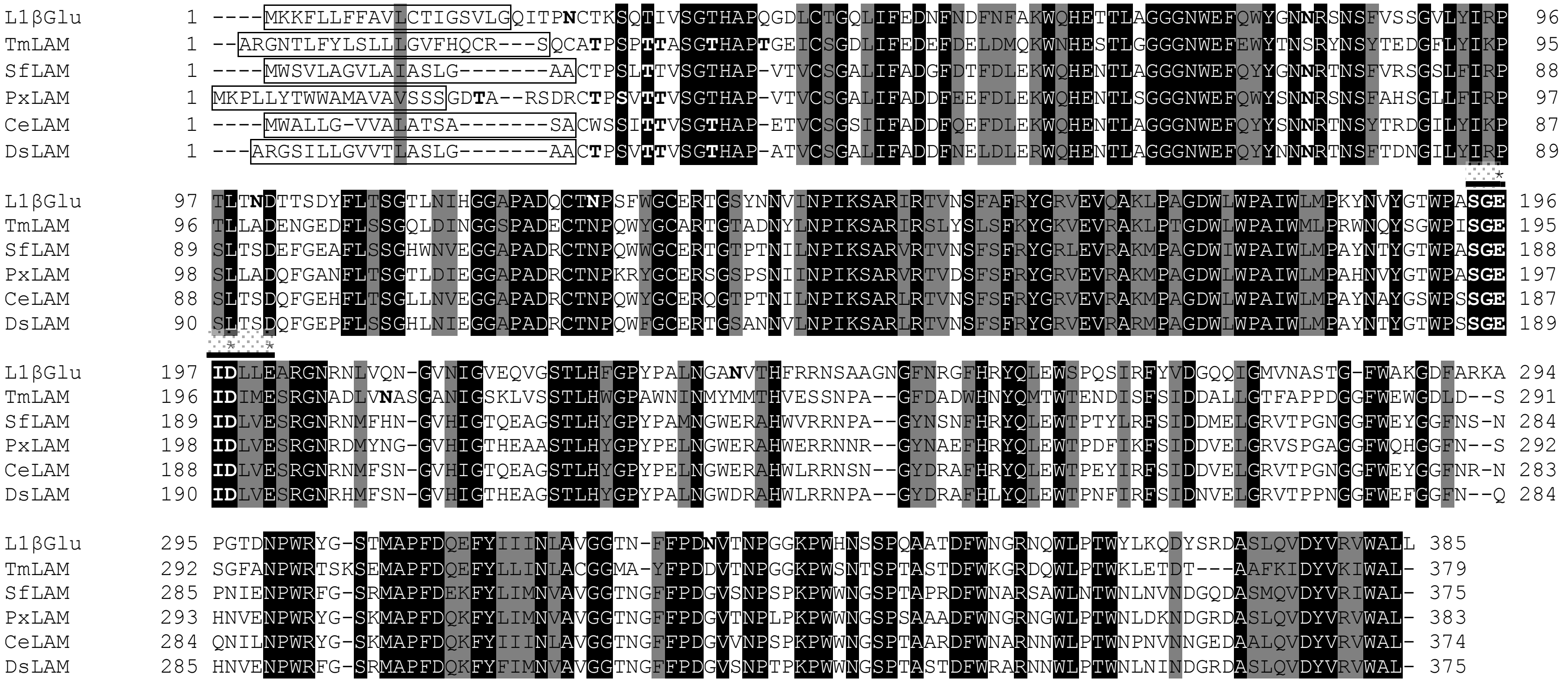

Figure S1. Amino acid sequence aligment of selected β-1,3-glucanases from different insect orders. Predicted signal peptides are boxed. Conserved residues are with black background and consensus alternative are shaded. Catalytic region is indicated with a dotted box and catalytic residues are marked with asterisks. The sequences used in the aligment were retrieved from *Tenebrio molitor* (TmLAM: accession number ACS36221), *Spodoptera frugiperda* (SfLAM: ABR28478), *Plutella xylostella* (PxLAM: ACI32820), *Colias eurytheme* (CeLAM: ACI32831) and *Diatraea saccharalis* (DsLAM: ABR28479). The sequence NSFM-140g04 retrieved from *Lutzomyia longipalpis* EST library is named as L1βGlu.
