# Supplementary material for: Expression pattern of glycoside hydrolase genes in Lutzomyia longipalpis reveals key enzymes involved in larval digestion
Source: Front Physiol. 2014 Aug 5;5:276. doi: 10.3389/fphys.2014.00276 (PMC4122206; doi:10.3389/fphys.2014.00276)
Supplement: Supplementary file 2 [file DataSheet2.PDF]

```
LlGBP1       ----------------------------------------------------------------------------------------------------
LlGBP2    1  -------MWTNVLLLFLCASAVVSYRIQSPIVEVC-PQALRVSIPDSPGVQLFAFHGNINHPIGETEAGLMSKDVTRKTAGRWVFETTAESFTLGDTINY  92
AsGRP     1  MRHLLLPLVIVFLSAGISYGKIKQFSIPDVEIEFLKPRGFRASIPGVPGIKLFAFHARINKKFEQFEEGDFADDITEPSEDGRWAFQTTKANIANDQILH 100
TmGRP     1  ------MKVLVVFIFCLVRSTFGQFEVPDALVEVFRPRGLRVSIPDQEGIKLFAFHGKINEEMNGREGGTFSRDILKAKNGRWTFYDANARLKEGDILYY  94
BmGRP     1  --------MYKTCVWVLLFKIVLCYEAPPATLEAIHPKGLRVSVPD-EGFSLFAFHGKLNEEMEGLEAGHWSRDITKPKNGRWIFRDRNAALKIGDKIYF  91
DmGNBP    1  MRWEFLPCLLLLISN----NKIFGFKVPSINFEMLKDEGFEVSIPDEPGIQRVFYMFQIDDTCP-----ALMDYITEAVNGSWVSKQKMSLQNNDKLQIS  91

LlGBP1       ----------------------------------------------------------------------------------------------------
LlGBP2   93  WIYVQHR----GLGYRSDVMKYTIN--------------------------AFSNRCEAYEVDSSEE-------------EVQVETQKFGRRRIKGENS- 148
AsGRP   101  YWVY----------VQHEDRSYWLTNQKQRKN--------------AAAKTTTTTTTTAKPKTTKKTNEKGKTTTAEPCVPTVTTVNGKASCSGRMIF 176
TmGRP    95  WTYVDYFDGKNKLGYPNDDQKFVVKQLLDKDG----------------AAPSVTPPTVTKAPPQEHTTLESG-------CKASVTTK-VNERVCAGEQIF 170
BmGRP    92  WTFV----IKDGLGYRQDNGEWTVEGFVDEAGNPVNTEGSEITPGVEFTSTSLNPESPQSIPNQPPDNLPAKPPSEGYPCELSLSTVSVPGFVCKGQLLF 187
DmGNBP   92  MLVQ----------FNEEIFEKSETRVIINTR--------------LLTTKDSSSRGITFLTGEGECQAYLAPAQQAKRCKAAQTIVSNGRHTCQGELIF 167

LlGBP1    1  ----------------------------------------------------NWKIDFGHKCTP---------STIVNNDIEC-VRQGGGRQIIPPVVS  37
LlGBP2  149  ------------------------------------SHYQHHYHHHHHHEDEGNRRFGHGHYG-----------------------WRPPTFPSAPSFPA 189
AsGRP   177  EDTFQTLDLQK--WQREIRIPLDTESAEFVSYQDSPENSFVVGGHLFIVPTLLNMNPDFSEERIRTGELVLSGCSSPTNNEYEC-RRKAALSTILPPVVS 273
TmGRP   171  HEDFTT--FETNIWRPEVKFAD-KPDYEFVFYRAGPPNLQVKHHRLTIRPVPSDAVFGEGFVSRREKVNLAPACTGVHG-SIEC-VQTPGAFLILPPVTS 265
BmGRP   188  EDQFNIPIHRGKIWVPEVKFPG-EPDFPFNVYLS--DNAEVNDGKLIIKPATLESKYGEDFV--RQSLDLSERCTGTVG-TAQC-LREASGPLILPPIIT 281
DmGNBP  168  EDNFSEAQLNKTTWKHDIRQRMYHVEEELVAFDDAARNCFVKEGELHIVPTIATEVTDGSFKLGDR-------CTAVESPEQECNIAHGIFYSIKPPVFS 260

LlGBP1   38  AQLTSKNHFRFRYGKIVVRAKLPKGDWLFPEIWLKSSQNVYGDENFVNGLMRVAFIRSNEYLTSADGREIDGLYLRGMLVLDRGETNRERWMKTEVG-QR 136
LlGBP2  190  PPNYPKPPTPFPFPTGPSGVCFCDKEIQKVNEKLSETRQQLQGAINATERIKEDFESLNDVLGQ---------------MLEKLNYGRKLLLTGVLP--- 271
AsGRP   274  AKLNTKHSFHFRYGRVEIRAKLPKGDWIFPQLLLQPNDNYYGYADLASGMLLVAHVLSNEQLIAQDGTRVDGHRLRGGAVLTAKAQLRNAFLKTNML-DE 372
TmGRP   266  AQISTKGKWSFKYGKVEIRAKLPKGDWIYPELYLNPVNEEYGPG-YASGQIRIAFSGGNEDLCR---------DLRGGCILGSRPAARNYAVKNIVKNSG 355
BmGRP   282  AKISTRHQFAFKYGRVEIRAKMPKGDWLYPEILLEPRDNIYGVRNYASGILKIASVKGNAEFSK---------KLYAGPIMTGSDPYRSFYLKENIG-YE 371
DmGNBP  261  AQIHTRNSFSFKFGKIVVRAKLPKGDWLFPYLMLQP-VSTYAETHYAK-QLRIAYARGNANLRTKQGDDISGNHLYGGGVVWHHGN-AVQFLKDKIS-NS 356

LlGBP1  137  HWGSDFHEYTLTWTDEMISMAVDGRPYAHFREKFCDTNSTKSCGVAHANVWENGQPPMAPFDQEVYVVLGVGVAGLSDFPEGCLT-TNRHVKPWRNTDPR 235
LlGBP2  272  ---PGDNPYELIRTILTDKLDLDDLKLRILNAT------------------------TTVHGAILFEMTSTTDKFRILLRSKRLE---RSKIRIINYQD 340
AsGRP   373  HFSDNFHTYGLVWKPDSIALTVDGFQYATLRDQFKTYGVAN--NLTQANLW-NADNVMSPFDQEFYLSLGVGVGGIADFPDCSLTGSLKQPKPWNNTSPK 469
TmGRP   356  SWSDDFHKFIVIWKPDQITMMVDDQVYGNIYPPEGGFVSEAYNLDLVNVERWRGGTSFAPFDKEMYLVLGVGVGGHCFEDRSDAT------KPWTNNDPK 449
BmGRP   372  SWNNDFHNYTLEWRPDGITLLVDGESYGEIKPGEG-FYNVANSYKVEAAPQWLKGTIMAPFDELFYVSIGLNVAGIREFSEDISN------KPWKNSATK 464
DmGNBP  357  HYGDDFHNYTMIWQRDKITLMVDDEVYGELYDG------------------------LPFFNEKCFIIFGVTVGGFLNFDDSLLA---KDVKPYKNREPR 429

LlGBP1  236  AEWFFYRDKDNWYPTWKGEDSGLQVDWVRVYSL 268
LlGBP2  341  DENEIFPVSSSPAPIIDGPDPAIDVRFGES--- 370
AsGRP   470  AEYFFYQNRNVWYRTWT--DPELKVDYVRVYAL 500
TmGRP   450  SQKKFYQAAAQWGATWS-NASRLEVDYVKVSAL 481
BmGRP   465  AMLKFWDARSQWFPTWD-EDSALQVDYVKVFAI 495
DmGNBP  430  AALSFWQHRDAWAPTWG-RHSAMVIDYVRVYAE 461
```

Figure S2. Amino acid sequence aligment of selected β-glucan binding proteins (βGBP) from different insect orders. Predicted signal peptides are boxed. Conserved residues are with black background and consensus alternative are shaded. The sequences used in the aligment were retrieved from *Armigeres subalbatus* (AsGRP: accession number AAT99011.1), *Tenebrio molitor* (TmGRP: BAC99308), *Bombyx mori* (BmGRP: BAA92243) and *Drosophila melanogaster* (DmGNBP: AAF33850). The sequences NSFM-111b04 and NSFM-14b06 retrieved from *Lutzomyia longipalpis* EST library are named as LlGBP1 and LlGBP2, respectively.
