# Supplementary material for: Expression pattern of glycoside hydrolase genes in Lutzomyia longipalpis reveals key enzymes involved in larval digestion
Source: Front Physiol. 2014 Aug 5;5:276. doi: 10.3389/fphys.2014.00276 (PMC4122206; doi:10.3389/fphys.2014.00276)
Supplement: Supplementary file 3 [file DataSheet3.PDF]

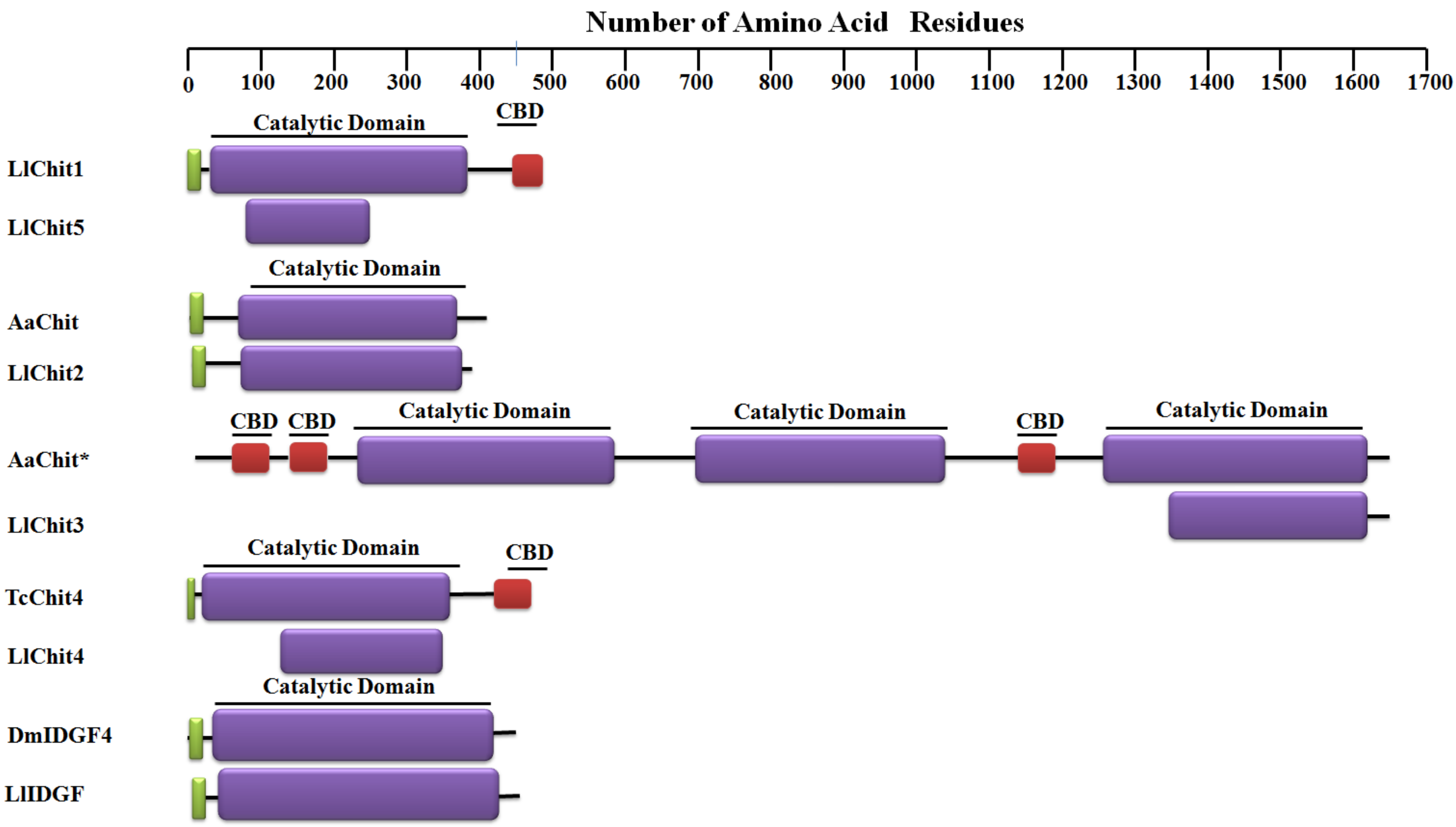
Number of Amino Acid Residues
0
100
200
300
400
500
600
700
800
900
1000
1100
1200
1300
1400
1500
1600
1700
Catalytic Domain
CBD
LlChit1
LlChit5
Catalytic Domain
AaChit
LlChit2
CBD
CBD
Catalytic Domain
Catalytic Domain
CBD
Catalytic Domain
AaChit*
LlChit3
Catalytic Domain
CBD
TcChit4
LlChit4
Catalytic Domain
DmIDGF4
LlIDGF

Figure S3. Schematic diagram of the domain archicteture of chitinases and chitinases-like proteins obtained from *L. longipalpis* EST library. Signal peptide, catalytic domain and chitin binding domain (CBD) are boxed with green, blue and red background, respectively. Black lines indicate linker regions. The domains were outlined using as reference: *L. longipalpis* chitinase (LlChit1: accession number AAN71763.1), *A. aegypti* chitinase (AaChit: XP_001661690), *A. aegypti* chitinase (AaChit* AAB81850), *T. castaneum* chitinase 4 (TcChit4: NP_001073567) and *D. melanogaster* imaginal disk growth factor 4, isoform A (DmIDGF4: NP_511101). The sequences NSFM-154b12, NSFM-88d12, NSFM-24g06, NSFM-96h07 and NSFM-18f06 from *Lutzomyia longipalpis* EST were named as LlChit2, LlChit3, LlChit4, LlChit5 and LlIDGF, respectively.
