# Supplementary material for: Expression pattern of glycoside hydrolase genes in Lutzomyia longipalpis reveals key enzymes involved in larval digestion
Source: Front Physiol. 2014 Aug 5;5:276. doi: 10.3389/fphys.2014.00276 (PMC4122206; doi:10.3389/fphys.2014.00276)
Supplement: Supplementary file 4 [file DataSheet4.PDF]

```
LlChit2     1 MR-LDIELIVILSLISIASGTLSPSKGQ-KGSKNAELKVRKGP--IDTTREHLIREEPSAKEILVENGAYYRNTSLRNFKGTVLGYVTPWNNYGYDIAKT  96
CqChit      1 MKFFVGVVLVALATVQLTGATLSPSDIKNKGKKVKELKTKQGPQATSVFERGLVQQEPSAKDILVENPAYHEETSLKNFNGKVLGYVTPWNNHGYDVAKI 100
AaChit      1 MRQIFAFLALLLALVQISGGTLTPSDVKGKGKKVKELKVKQGPQPSSVFDRGLIQEEPSAKDILVESGAYYEETALKNFKGTVLGYVTPWNNHGYDVAKI 100
BtChit      1 MN-IFTLIISVLLFIELSNCTLSPPKNRNSKKSKGKIEIKKGPVGQDVFSRILVVKNPKTHDIIRESGFYFSNTTRRRFTGEVLGYITPWNNNGIEVSKI  99
                                                                                                        CR2
LlChit2    97 FSPKFDVVAPIWLQILRKGDKQYELAGMHDVDEQWMKDVKRN-SGFKTK-----ITPRVLFDKFKDEDFSKLLSYPEERKIVSRIILNACDTYRFDGIVL 190
CqChit    101 WGSKFNYVSPVWLQVLRKGPKQYELGGAHDIDAGWVKDVKKAGQSIGNK-----VVPRILFDKFTDKDFSQLLTYSEERTVAARLIVDTVRRYRFDGIVL 195
AaChit    101 WGGKFNYVSPVWLQVLRKGPKQYELGGAHDIDAGWVKDVKKAGEAINNKGKCTFFVPRVLFDKFTDKDFSQLLTYSEERTIAAKLILNTVRKYKFDGIVL 200
BtChit    100 FHGKFTMVSPVWLAFPKGNTSTYKLL-THDVQKKWLKEMKATNNENHHVK----ILPRVLFEHWSVNDIVGLYSDTYKQTTLLSALVDTAKNFHFDGYVL 194
              CR2                                                           CR3
LlChit2   191 EVWATLALR-MDDPFLYDLVIDISKPLLEQKKLVYMAIPPRRDHLYDLFTQENFDYLFDYVSGYCVMAYDYSNAQRPGPNAPIKWVRDTVEFFCPSTSPN 289
CqChit    196 EVWSQLAAR-VDDEFLVGLVREICQTLTEGGFECILVIPPARKETYDLFSKRHFETLVPVVSAFSLMTYDYSTVQRPGANGPLYWVKNAVQHICPDSAEN 294
AaChit    201 EVWSQLAAR-VDDEYLVGLVKEICETLTGANFACILVIPPARKETYDLFSRRHFESLVPVVTAFSLMTYDYSSIQRPGACAPLYWVKNAVQHICPDTAED 299
BtChit    195 EIWNQFVLTGVDTQIVVALIQFIAHQLKNNNLDTILAVPPSRGPNVQLFNKDQFDQLSPYIKAFSLMTYDYSSIQRPGPNSPLNWARECVELLVPEKGP- 293

LlChit2   290 YREKRKKILLGLNMYGNDFTPEGGGPIVGHEYLRLLKYVKGRLQHDEKDAENFFEVKTPTGRHIVFYPTLYSIKKRIELAQELGTGLSLWELGQGLDYFY 389
CqChit    295 LKEKRAKILLGLNLYGSDYTPNGGQPIIGHEYLALLKHLKGHLTFDEHDVENFFEVKTSNGRHMVFYPTLFSINERLKLARELGTGISLWELGQGLDYFY 394
AaChit    299 VKEKRAKILLGLNLYGSDYTPNGGQPIVSHEYLALLKHLKGHLTFDDHDVENFFEVKTSNGRHMVFYPTLFSINERLKLAKDLGTGISLWELGQGLDYFY 399
BtChit    294 ---KRAQILLGLNLYGYNYTPEGGGAILGLDYLKILESFKGKIQWDDKSKEHFFESKSPGGSGIIFYPTLYSILHRLDLAAELGTGISIWELGQGLHYFY 390

LlChit2   390 DLL 392
CqChit    395 DLF 397
AaChit    400 DLF 402
BtChit    391 DLL 393
```

Figure S4. Amino acid sequence aligment of selected insect chitinases similar to L. longipalpis NSFM154b12 (named as LlChit2). Predicted signal peptides are boxed. Conserved residues are with black background and consensus alternatives are shaded. Catalytic residues are marked with asterisks and the conserved regions (CRs) are indicated with dotted boxes. The sequences used in the aligment were retrieved from *Culex quinquefasciatus* (CqChit: accession number XP_001869617), *Aedes aegypti* (AaChit: XP_001661690) and *Bombus terrestris* (BtQuit: XP_003399293.1). The sequence NSFM-154b12 retrieved from *Lutzomyia longipalpis* EST is named as LlChit2.
