# Supplementary material for: Expression pattern of glycoside hydrolase genes in Lutzomyia longipalpis reveals key enzymes involved in larval digestion
Source: Front Physiol. 2014 Aug 5;5:276. doi: 10.3389/fphys.2014.00276 (PMC4122206; doi:10.3389/fphys.2014.00276)
Supplement: Supplementary file 6 [file DataSheet6.PDF]

```
LlChit4        --------------------------------------------------------------------------------------------------
LmChit       1 -MSHFLLGLSLLLGLSLSTYGAEDKIVVCYHGSWSAYRNGNGRFEIEDIQPELCTHLIYTFVGITSSGEVRILDEWLDLASG-KNAYNRFNALKSSST 96
TcChit4      1 ---MMLKGLALLS--ILGIVSAATNKVVCYHGIWSTYRLNNGKFTVEDIDPTLCTHLIYSFVGLGDDSRIKHLEPNLDVNQG-NLKKFNALKLKNPNL 92
AgChit       1 MTRFVPLWSILLVAACSLLAAVESREVVCYYGTWAVYRQNGGGFDINNINPALCTQLVYAFFDVGPDGSIVPSDATVASGQYNMLAKFSDLKQRYPAL 98
                  CR1                                                       CR2 *

LlChit4      1 -----------------------------VSEVV----------------HPR-----TGRLSS--ELLKDLRTRFNSEGLILTTAVGASAHFLSSSY  46
LmChit      97 KTLVAIGGWNEGSATYPAVMNDASLRAKFVQNVVNFVKTYGFDGFDLDWEYPANRGGSPGDLTAFVSLIKELRAEFDKHGYLLTAAVGVGRYLVGSAY 194
TcChit4     93 KTLVAIGGWNEGSVNYSKMAASSSLRAVFIKSVVEFVKTWGFDGFDLDWEYPGQRGGAYNDKSNYVTLIKEMRKEFDKNGLILTAAVAAASGSVDISY 190
AgChit      99 KTIAAVGGWSDTDN-FTPMATNAQRRATFVRSAVALLQKNRFDGLDVDWEYPT-------DKGVFVQLLRDLAAAFAPSKYLLTVAVGGTSFEAINRY 188
                              CR3

LlChit4     47 NAAEMAKYCHYILLMTYDLRSAYDGATGQNAPLYAS--SKESSGVS-TYNVDAAVKAWIGAGADPARIVLGIPLYGKSFTLASSATNGLGARTTGPGP 141
LmChit     195 DVPQISQYLDFINLMTYDLHGSWDGKTGQNAPLYAS--SADKTEAERQLSVDSSVRYWIENGADPSKLVLGMGTYGRTFTLASTANTGVGAPATGPGT 290
TcChit4    191 DVPALSKYLDIINVMAYDLRGSWDGVTGHHSGLYPS--AVDTTTNQKLLTVDAAIRGWIQRGADPQKIALGLPVYGKTFTLSSASNAKLGAPVKGAGN 286
AgChit     189 DIPAIASIVNFINLMTYDLQGDYG-VTRHQAALYPGSAALDNSDYKRALNAEAVITYWLSKGAPASKLNLGIPLYGRTFKLANPSVDGVGAPVSGVGT 285

LlChit4    142 IGPYTQDPGTLNYIEICEKQQQGGWTTVWDNDQKVPYTYKGTEWIGYDNVESVKIKS----------------------GLC---------------- 201
LmChit     291 NGPYTMESGMMGYNEICEKINAGGWTVTWDDEQKVPYAVSGNQWVGYDNEESIRLKSQYVLDMGLAGGMIWSLETDDFKGLCGSKTYPLLSTINEVLR 388
TcChit4    287 SGKYTGEAGMLGYNEIVELQKEGGWKVVWDDTQKNTHMYKGDQWVAFDSPKAISNKVEYAKSLNLGGVMIWSIETDDFRGVSG-TKYPILKAIHQTLG 383
AgChit     286 KGPITQEAGILAYYEICSSPTLT--RKQYDSAQVGAFASGGGEWVSYDSVESIGQKCNVIAKYGLGGGMVWAIDMDDFAGKCG-SKFPLMTALNNCVN 380

LlChit4        --------------------------------------------------------------------------------------------------
LmChit     389 GITSTKTSSSVSSSPSSSSSSSGSSG-----SSGSSSSSAGSSSGVCSSEGYTRDPSDCSVFYLCSAAAGGFVASKFTCPGDLVFDASSSACKYRSLV 481
TcChit4    384 GSSNEIVEP---VPQPVEEVTQKPSS-----TKAPAPPSRDLTTTLCTKAGYVRDPDDCSIFYYCLAYNGGFVPLEQRCNAGLVFDEEKLWCDYPEVV 473
AgChit     381 RNGAVALAPAPTPAPTTARPATATTARQIVTTTTTKPVTPSSGVFVCPRDGYFRDPRNCAKFYRCYDGGR---QALFDCPSGLYFNEAITACDWPYNV 475

LlChit4        --
LmChit     482 AC 483
TcChit4    474 KC 475
AgChit     476 KC 477
```

Figure S6. Amino acid sequence aligment of selected insect chitinases similar to *L. longipalpis* NSFM-24g06 (named as LlChit4). Predicted signal peptides are boxed. Conserved residues are with black background and consensus alternatives are shaded. Catalytic residues are marked with asterisks and the conserved regions (CRs) are indicated with dotted boxes. The sequences used in the aligment are from *Locusta migratoria manilensis* (LmChit: accession number ABK76337), *Tribolium castaneum* (TcChit4: NP_001073567) and *Anopheles gambiae* (AgChit: XP_315351).
