# Supplementary material for: Expression pattern of glycoside hydrolase genes in Lutzomyia longipalpis reveals key enzymes involved in larval digestion
Source: Front Physiol. 2014 Aug 5;5:276. doi: 10.3389/fphys.2014.00276 (PMC4122206; doi:10.3389/fphys.2014.00276)
Supplement: Supplementary file 7 [file DataSheet7.PDF]

```
LlChit5      1 ----------------------------------------------------------------------------LGGSIETDDFRGFGGRGTFPLLKE  24
LlChit1      1 --------MKTLVFLCVALSILGLAVTEKKIVCYHGTWSYYRQGNGKFGVAQIDPFLCTHLVYTFFGISSEGGIRILDPYLDLDENYGLGNIRKFNELKK  92
OnChit       1 -------MGLILLFVLGFCASAVFANDDKIVVCYYGTWATYRTGLGKFDVDDIDPFLCTHLVYAFIGINAEGTALALDPELDVE----RGNFKNFTSLKE  89
DmChit8      1 MWSVSGLVKLLLGVILMAASSSAQGNSSKNVVCYQGTWSVYRPGLGKFGMEDIDPFLCTHLIYAFLGIEETGQLRVIDAYLDLEENSGRGNIKSFNALKL 100
                      CR1                                          CR2 *
LlChit5     25 LNASLLGNNVSTSNETDPEIMSLLSAERKK----TAALKKFIHDFFFKTFQPKYQKLIQFNLLKEKYFSESFTELYGTSPQMLSDT-------------- 106
LlChit1     93 VNPKLKTIAGVGGWNEGSVTFSQVVNDPRKRQNFVKNSLEFLKKYNFDGLDVDWEYPAQRGGNQEKD-KEAYTLLLKELSEFLHPKGYSLSAAVASAEFS 191
OnChit      90 KNPNLKTLVAVGGWSEGSANYSIMAAEPEYRQNFINTSLAMILEYNFDGLDVDWEYPNRRDTVHGEDDIENFSTLLKELREEFDNYGLLLTVAVAAVEEA 189
DmChit8    101 KNPVLKTLVAVGGWNEGSKRFSLVARDPSKREKFVDDVVRFLQRHGFDGLDLDWEYPGQRHSLDNED-RSNYITFLKELKEGLEPFGFILSAAVGSAQFS 199

LlChit5    107 --------------------------------------YKREKDL------------------------------------------------------- 113
LlChit1    192 AKISYNIAEVSKYLDFIGVMTYDLHGSWDPKIGNNAPLYAGSWEQTELE---KQLNVDAAIKYWLSNGGAPEKLLLGVPLYGRGFRMVNGQ-NKPGSVHG 287
OnChit     190 AVQSYDVPSVAKYVDYIGVMTYDMHGAWDSVTGHNAPLFISEGESAENE--STLYNVNNAVQYWLSAGCPPEKLVMGVPFYGRTFNLSDPSVNAPNSPSN 287
DmChit8    200 AEISYDIPAMVPYLDLINVMAYDLHGPWDQVVGINAPLYAAEKDASDSSGRQQQLNVDAVVKYWLKAGAPAEKLILGVPFYGRSFTLATAEGNQPGAPHI 299

LlChit5        ----------------------------------------------------------------------------------------------------
LlChit1    288 GPCQAGPYTQTPGMMGFNELCEKRRNEK-WIDFWDDEQFVPYSTKNDQWIGFDDEKSIKFKSNYVNSHNLGGVIVWSIETDDFRGFCGRGTFPLLKELNA 386
OnChit     288 GAGLAGPYTAESGFIGYNEFCYILQNESSWTVQTDNLAKVPYAFLDYNWVSFDNVESMTAKVEYANSFNLRGIMLWSIETDDFHGLCGEGTFPLLNTINT 387
DmChit8    300 GKGIAGNYSREPGVLGYNELCEMMEREE-WTQKWEATQQVPYAYRQRQWVGYEDPRSLALKAQYVMDNHLGGIMIWSLESDDFRGTCGQQPYPLLHEINR 398

LlChit5        ----------------------------------------------------------------------------------------
LlQuit1    387 SLLGNNHTWTPPSTSTTEWNPNPNVPQANHHQSSQVLYVLKWLLWGSQMTAVSLHQRINGTQYTFFCPHGLVFDPAIIACNWPHIVQC 474
OnQuit     388 VLAEG-------STEARHNNPP--------------------------------------HHHHH----------------------- 407
DmQuit8    399 VLFGGNTPSGL-TTESNRESPSEGFSCPADAPAG---------YIRDPDNCSKFYYCSGGKTHNFDCPSGLNFDLDTKSCNYSGSVKC 476
```

Figure S7. Amino acid sequence aligment of selected insect chitinases similar to *L. longipalpis* NSFM-96h07 (named as LlChit5). Predicted signal peptides are boxed. Conserved residues are with black background and consensus alternatives are shaded. Catalytic residues are marked with asterisks and the conserved regions (CRs) are indicated with dotted boxes. The sequences used in the aligment are from *Lutzomyia longipalpis* (LlChit1:accession number AAN71763), *Ostrinia nubilalis* (OnChit: ADB85578.1) and *Drosophila melanogaster* (DmChit8: NP_611542).
