# Supplementary material for: Expression pattern of glycoside hydrolase genes in Lutzomyia longipalpis reveals key enzymes involved in larval digestion
Source: Front Physiol. 2014 Aug 5;5:276. doi: 10.3389/fphys.2014.00276 (PMC4122206; doi:10.3389/fphys.2014.00276)
Supplement: Supplementary file 8 [file DataSheet8.PDF]

```
LlIDGF       1 -MSSLTIYLAVALASLVAIAYGQQGTSKVICYYDSRSFVREGLGKTNLADIEPALPFCTHLVYGYAGINGGTNKLVSLNENHDLDQGKGFFRQVTTLKRK  99
DmIDGF4      1 --MKLYALFSLLVGSLAIGQISAAGSHHLLCYYDGNSFVREGLSKLILTDLEPALQYCTHLVYGYAGINPSSNKLVSNNEKLDLDLGSSLFRQVTGLKRK  98
TcIDGF2      1 -MGSFKPLLFFAVATLFAAYCKAES--KVVCYYDSKSHFRQGQAKFEITDLEPALQYCTHLIYGYAAIDEETYKLTPLNEQFDVIKDN--YRKVTDLKKR  95
CqIDGF       1 MRLIQAGLLAVVLVAFLVHGSHGQTTPKVLCYYDGANFLIEGLAKVSLTDIEAALPFCTHLVYGYAAIDPTSNKAVSKNPTLDLDTGKSNYRLVTQLKRK 100
                   CR1                                                  CR2 #

LlIDGF     100 YPALKILLGVGGG------ADPESSQYLSLLESSAGRISFINAAYTLVKTYDFDGLDLSWQFPVVKPKKIRGTFSSFFHKIKKPFTSDDVIDEKSEEHKE 193
DmIDGF4     99 YPALKVLLSVGGDKDT---VDPENNKYLTLLESSNARIPFINSAHSLVKTYGFDGLDLGWQFPKNKPKKVHGSIGKFWKGFKKIFSGDHVVDEKAEEHKE 195
TcIDGF2     96 FPKLKVLLSVGGNADVSGQDEEKNIKYRNLLETTTRRLAFVNSAYTLVKAYGFDGLDLAWEFPENKPKKIRSKLGSIWHSVKKTVAGDKVLDENAAEHRE 195
CqIDGF     101 FPALKVLLGVGGYR-----FSAPSPKYLELLESGAARITFINSVYAIVKAYEFDGIDLAWQFPQNKPKKIRGTTSKLWHGFKKVFSGDSVLDEKADEHKE 195

LlIDGF     194 EFTALVRELKNSFRHDNYQLALTVMPNVNSSLYFDVPAIINNLDYVSLAAFDFQTPDRNPKEADYPAPLYELPERNPESNVNYQVQWWLGQRGPASKIIV 293
DmIDGF4    196 AFTALVRELKNAFRPDGYILGLSVLPNVNSSLFFDVPAIINNLDYVNLHTYDFQTPERNNEVADFPAPIYELNERNPEFNVNYQVKYWTGNRAPAAKINV 295
TcIDGF2    196 QFVSLVRELRGAFKAENLLVTLTVLPNVNSTVYYDPRALSPNLEFVVLEAFDFYTPARNPKEADYPSPLYELVDRRNDENIDAQVKYWMSNGAPSAKIVL 295
CqIDGF     196 EFTALLRELKNAFRADGYQLGITVLPHVNATMFMDIPAIINYLDFVNIEAVDMQTPERNPKEADYVAPLYELTDRVPGNNVDGLVKVWLGANTPPSKIVV 295

LlIDGF     294 GIPTFGHCWKLGEDSGITGVPPLRTPKEGHPEGPQLKVPGLVSYPEACSKLTNPSNAHLKGEDAPLRKVNDPTKRFGNYAFRLGDPDRDYEHGMWCSFED 393
DmIDGF4    296 GIATYGRAWKLTKDSGLTGLPPVAEADGVAPAGTQTQIPGLLSWPEVCAKLPNPANQHLKGADGPLRKVGDPTKRFGSYAYRSADDSG--ENGVWVGYED 393
TcIDGF2    296 GLPTFGRAWAMDDESDINGTPPL-HISGPAEAGPLTKDAGLLSYPEICTLLNDPQNAKTQKA-FQVKRVPDPSKRKGSYIFRLPDENN--NGGFWVGYED 391
CqIDGF     296 TIPTHGRGWKMNADSGITGVPPL-TADGTGPAGPQLQQEGYYTWGETCAMLPNPSNTALKGAQAPLRKVGDPTKRFGSYAFRLPDADG--ENGLWVSYED 392

LlIDGF     394 PDTAGNKAGYVRAKGLGGIGLFDITLDDFRGTCSG-DKFPILRAAKYRL 441
DmIDGF4    394 PDTAAIKAEYVKREGLGGIAVVDLSFDDFRGGCTGHDKFPILRQVKSKL 442
TcIDGF2    392 TDTAGYKASYVKAKGLGGIAIVDLSLDDFKGTCGR-DKYDILRTAKTHL 439
CqIDGF     393 PDSAGNKAAYVKAKGLGGIGINDLSYDDFRGTCAG-EKFPILRAAKYRL 440
```

Figure S8. Amino acid sequence aligment of selected insect chitinase-like proteins similar to NSFM-18f06 (named as LlIDGF). Predicted signal peptides are boxed. Conserved residues are with black background and consensus alternatives are shaded. The conserved regions (CRs) are indicated with dotted boxes. The sequences used in the aligment are from *Drosophila melanogaster* (DmIDGF4: accession number NP_511101), *Tribolium castaneum* (TcIDGF2: NP_001038092) and *Culex quinquefasciatus* (CqIDGF: XP_001844673).
