# Supplementary material for: Expression pattern of glycoside hydrolase genes in Lutzomyia longipalpis reveals key enzymes involved in larval digestion
Source: Front Physiol. 2014 Aug 5;5:276. doi: 10.3389/fphys.2014.00276 (PMC4122206; doi:10.3389/fphys.2014.00276)
Supplement: Supplementary file 9 [file DataSheet9.PDF]

```
LlLysi       1 MCTTRWILGGLLVAIAISLGNADVSHVVSGNQ---TPLTDICLGCICEAMSGCNRAAACDG---NICGLFKITHPYWVDAGKPTQSADSPEATGAFANCV 94
DmLys        1 --MRVFLLYSIYLLLVLSP-----SLVQGQGHVLDKPVTELCLTCICEAISGCNATAICTSAEKGACGIFRITWGYWVDAGKLTVNGEHPDSEKAFINCA 93
AgLys        1 -MVPKSLLLVCLIAIGVSSVLADVSHIAPPQQQLEDPVTDVCLSCICEASSGCDASLRCSG---DVCGMFAITWAYWADAGKPVQQGDSPDSQNAYANCA 96

LlLysi      95 TEPFCAGRTVQNYMSKFGQDCNKDGVVDCTDYLSIHILGGYGCGGEIPAKFTNALNQCLYQASAFQNSFGK- 165
DmLys       94 NDPHCAADLVQNYMKKFNQDCNDDGEMDCHDYARIHKLGAYGCQADMPYNFQSVFEECIERYEDEGFE---- 161
AgLys       97 NEPYCAARTVQGYMRKFGQDCNGDGRIDC DHAIVHKLGGYNCKNAVPIVYQSKIDECI-QRKAIEYSAARQ 167
```

Figure S9. Amino acid sequence aligment of selected insect i-type lysozymes similar to NSFM-123b01 (named as LlLysi). Predicted signal peptides are boxed. Conserved residues are with black background and consensus alternatives are shaded. The sequences used in the aligment are from *Drosophila melanogaster* (DmLis: accession number CAA21317) and *Anopheles gambiae* (AgLis: AY659931).
