# Supplementary material for: Expression pattern of glycoside hydrolase genes in Lutzomyia longipalpis reveals key enzymes involved in larval digestion
Source: Front Physiol. 2014 Aug 5;5:276. doi: 10.3389/fphys.2014.00276 (PMC4122206; doi:10.3389/fphys.2014.00276)
Supplement: Supplementary file 10 [file DataSheet10.ZIP › Supplementary Tables/Table S1.PDF]

Table S1. Primers used for PCR-amplification of cDNA sequences of β-1,3-glucanase, β-glucan binding proteins, lysozyme, chitinases and Ribosomal protein 60S of *L. longipalpis* (Jacobina population, Brazil).

Protein	Primer Name	Sequence (5'-'3)	Fragment Size (pb)
β-1,3-glucanases/β-glucan binding proteins	14b06R	ACCATCACCATGAAGATGAGGGCA	312
	14b06F	TGCCGTAGTTGAGTTTCTCCAGCA	
	111b04R	TCATGCGGGTGGCCTTTATACGAT	462
	111b04F	AGAAGAACCACTCAGCACGTGGAT	
	LAMS2R	GCAGTGCCCATACACGGAC	542
	LAMF	GGAATTTGGTGCAGAATGGCGTGA	
Lysozyme	123b01R	TTGCCAAAGGAATTCTGGAAGGCG	218
	123b01F	TGTGTTACAGAGCCCTTCTGTGCT	
Chitinases	18f06R	TTCACCCAACTTCCAACAATGCCC	479
	18f06F	ACGGTCTGGACTTATCGTGGCAAT	
	88d12R	TGGCTTCTCTCATCGGCTGTTTCT	413
	88d12F	ACAGTCCATCCACCACTATTGGCA	
	24g06R	TCCTCATCCTACAATGCTGCCGAA	269
	24g06F	AGTCCATTGGTAGCTGAACTGGCT	
	96h07R	AGCTCAATGCATCCCTCCTTGGTA	228
	96h07F	GGGATGTACCGTAAAGTTCGGTGAAG	
	154b12R	ACCACCCTCGGGTGTAAAGTCATT	390
	154b12F	CGCTTTGATGGCATTGTCCTGGAA	
Ribossomal Protein 60S	RIBO60R	GGCTTGTGACACCCTTGAAT	750
	RIBO60F	TCTCATCGGAAGTTTTCTGC	
