# Supplementary material for: Expression pattern of glycoside hydrolase genes in Lutzomyia longipalpis reveals key enzymes involved in larval digestion
Source: Front Physiol. 2014 Aug 5;5:276. doi: 10.3389/fphys.2014.00276 (PMC4122206; doi:10.3389/fphys.2014.00276)
Supplement: Supplementary file 10 [file DataSheet10.ZIP › Supplementary Tables/Table S2.PDF]

Table S2. Number of cycles and set of primers used in multiplex PCR amplification of cDNA sequences of β-1,3-glucanase, β-glucan binding proteins, lysozyme, chitinases and Ribosomal Protein 60S of *L. longipalpis* (Jacobina population, Brazil).

Primer Set	Number of Cycles
Set 1: 11b04F, 11b04R, LamS2R, LAMF, 24g06F, 24g06R, Ribo60F, Ribo 60R	24
Set 2: 96h07F, 96h07R, 154b12F, 154b12R	35
Set 3: 123b01F, 123b01R, 18f06F, 18f06R	24
Set 4: 88d12F, 88d12R	35
Set 5: 14b06F, 14b06R	27
