# Supplementary material for: Expression pattern of glycoside hydrolase genes in Lutzomyia longipalpis reveals key enzymes involved in larval digestion
Source: Front Physiol. 2014 Aug 5;5:276. doi: 10.3389/fphys.2014.00276 (PMC4122206; doi:10.3389/fphys.2014.00276)
Supplement: Supplementary file 10 [file DataSheet10.ZIP › Supplementary Tables/Table S3.PDF]

Table S3. Similarity of sequences of GHF16, GHF18 and GHF22 members from *Lutzomyia longipalpis* ESTs library to selected glycoside hydrolases (best matches in Blast, nr database).

Clone	Hit Description	Accession Number	Identity (%)	E-value	Score
NSFM-140g04 (LlβGlu)	*Anopheles gambiae* str. PEST AGAP002798-PA (GNBPB2)	XM_312116	67	5e-166	347
	Aedes aegypti gram-negative bacteria binding protein	XM_001659746	65	8e-166	520
	Euphydryas aurinia beta-1,3-glucanase	JN248787	69	2e-161	382
NSFM-111b04 (LlGBP1)	*Anopheles gambiae* str. PEST AGAP006761-PA (GNBPA1)	XM_308984	44	3e-53	174
	Armigeres subalbatus beta 1,3-glucan recognition protein (GRP)	AY603183	43	1e-50	168
NSFM-14b06 (LlGBP2)	*Hepialus pui* beta-1,3-glucan recognition protein 4a (GRP4a)	HM459596	51	3e-23	118
	Locusta migratoria GNBP1	JF915523	49	4e-19	104
NSFM-96h07 (LlChit5)	*Lutzomyia longipalpis* midgut chitinase	AY148807	87	4e-29	136
	Phlebotomus papatasi midgut chitinase	AY644680	59	2e-15	90.9
NSFM-154b12 (LlChit2)	*Culex quinquefasciatus* chitinase domain-containing protein 1	XM_001869582	58	1e-143	375
	Aedes aegypti chitinase	XM_001661640	57	7e-143	364
NSFM-88d12 (LlChit3)	*Aedes aegypti* brain chitinase and chia	XM_001655923	79	2e-174	613
	Culex quinquefasciatus acidic mammalian chitinase	XM_001857818	79	4e-174	612
NSFM-24g06 (LlChit4)	*Locusta migratoria manilensis* midgut chitinase mRNA	EF090723	51	3e-72	165
	Culex quinquefasciatus chitotriosidase-1	XM_001869597	52	2e-68	158
NSFM-18f06 (LlIDGF)	*Musca domestica* chitinase-like protein Idgf4-like	XM_005188622	65	7e-174	409
	Glossina morsitans morsitans imaginal disc growth factor 4	DQ307196	64	1e-170	401
NSFM-123b01 (LlLysi)	*Papilio xuthus* mRNA for similar to CG6426	AK401219	58	2e-51	210
	Ceratitis capitata lysozyme-like	XM_004520243	57	7e-50	205
	Coptotermes formosanus clone CFSNI2898 I-type lysozyme	JX915870	50	6e-48	199
