# Supplementary material for: Expression pattern of glycoside hydrolase genes in Lutzomyia longipalpis reveals key enzymes involved in larval digestion
Source: Front Physiol. 2014 Aug 5;5:276. doi: 10.3389/fphys.2014.00276 (PMC4122206; doi:10.3389/fphys.2014.00276)
Supplement: Supplementary file 10 [file DataSheet10.ZIP › Supplementary Tables/Table S4.PDF]

Table S4. Sequences and accession numbers of GHF16 sequences from order Diptera used for the cladogram of Figure 1.

Specie	Identifier	Accession Number
Aedes aegypti	Aa_ps	AAL76017
Anopheles gambiae	AgGNBP	ACN38130
Actinophrys sol	AsGBP (outgroup)	BAG32349
Armigeres subalbatus	AsGRP	AAT99011
Culex quinquefasciatus	CqGNBP	AEQ27734
Drosophila melanogaster	DmGNBP	AAF33851
Glossina morsitans morsitans	GmGNBP	ABC25063
Simulium vittatum	SvGNBP	ACH56895
