# Supplementary material for: Expression pattern of glycoside hydrolase genes in Lutzomyia longipalpis reveals key enzymes involved in larval digestion
Source: Front Physiol. 2014 Aug 5;5:276. doi: 10.3389/fphys.2014.00276 (PMC4122206; doi:10.3389/fphys.2014.00276)
Supplement: Supplementary file 10 [file DataSheet10.ZIP › Supplementary Tables/Table S5.PDF]

Table S5. Identifiers and accession numbers of Insect GHF18 sequences used for the cladogram of Figure 2.

Aedes aegypti		*Anopheles gambiae*		*Drosophila melanogaster*		*Tribolium castaneum*	
**Identifier**	**Accession Number**	**Identifier**	**Accession Number**	**Identifier**	**Accession Number**	**Identifier**	**Accession Number**
AaCht2	XP_001657537	AgCht2	XP_315650	DmCht2	NP_477298	TcCht2	NP_001034516
AaCht5-1	XP_001656234	AgCht4	XP_315351	DmCht4	NP_524962	TcCht4	NP_001073567
AaCht5-2	XP_001656233	AgCht5-1	HQ456129	DmCht5	NP_650314	TcCht5	NP_001034524
AaCht5-3	XP_001656232	AgCht5-2	HQ456130	DmCht6	NP_572598	TcCht6	XP_967813
AaCht5-4	XP_001656231	AgCht5-3	HQ456131	DmCht7A	NP_647768	TcCht7A	NP_001036035
AaCht6	XP_001662588	AgCht5-4	HQ456132	DmCht7B	NP_647768	TcCht7B	NP_001036035
AaCht7A	XP_001650020	AgCht5-5	HQ456133	DmCht8	NP_611542	TcCht8	NP_001038094
AaCht7B	XP_001650020	AgCht7A	XP_308858	DmCht9	NP_611543	TcCht9	NP_001038096
AaCht8	XP_001663097	AgCht7B	XP_308858	DmCht10A	EAA46011	TcCht10A	NP_001036067
AaCht9A	XP_001656054	AgCht8	XP_316448	DmCht10B	EAA46011	TcCht10B	NP_001036067
AaCht9B	XP_001656054	AgCht9A	XP_307732	DmCht10C	EAA46011	TcCht10C	NP_001036067
AaCht10A	XP_001655973	AgCht9B	XP_307732	DmCht10D	EAA46011	TcCht10D	NP_001036067
AaCht10B	XP_001655973	AgCht10A	XP_001238192	DmCht11	NP_572361	TcCht10E	NP_001036067
AaCht10C	XP_001655973	AgCht10B	XP_001238192	DmCht12	NP_726022	TcCht11	XP_974461
AaCht10D	XP_001655973	AgCht10C	XP_001238192	DmIDGF1	NP_477258	TcCht12	XP_972802
AaCht11	XP_001654045	AgCht10D	XP_001238192	DmIDGF2	NP_477257	TcCht13	NP_001036034
AaCht12	XP_001663568	AgCht11	XP_310662.5	DmIDGF3	NP_723967	TcCht14	XP_973005
AaCht13	XP_001655071	AgCht12	XP_316142.4	DmIDGF4	NP_727374	TcCht15	XP_973077
AaCht14	XP_001663099	AgCht13	XP_314312.4	DmIDGF5	NP_611321	TcCht16	NP_001034515
AaIDGF1	XP_001660745	AgCht16	XP_319801.4	DmIDGF6	NP_477081	TcCht17	XP_972719
AaIDGF2	XP_001660748	AgCh23	XP_001688641			TcCht18	XP_973161
		AgCht24	XP_316256.4			TcCht19	XP_973119
		AgIDGF2	XP_001237925			TcCht20	XP_970191
						TcCht21	NP_001034517
						TcCht22	NP_001038095
						TcIDGF2	NP_001038092
						TcIDGF4	NP_001038091
