# Supplementary material for: Expression pattern of glycoside hydrolase genes in Lutzomyia longipalpis reveals key enzymes involved in larval digestion
Source: Front Physiol. 2014 Aug 5;5:276. doi: 10.3389/fphys.2014.00276 (PMC4122206; doi:10.3389/fphys.2014.00276)
Supplement: Supplementary file 10 [file DataSheet10.ZIP › Supplementary Tables/Table S6.PDF]

Table S6. Identifiers and accession numbers of Insect GHF22 protein sequences used for the cladogram of Figure 3.

Specie	Identifier	Accession Number
Aedes aegypti	AaLys	CAC19819
Anopheles darlingi	AdLysC	AAB61345
Anopheles gambiae	AgLys	AAC47326
Anopheles gambiae	AgLys-1	AY659931
Anopheles gambiae	Ag-Lys2	EF492429
Culex tarsalis	CtLys	ACJ64375
Drosophila melanogaster	Dm	AAF57939
Drosophila melanogaster	Dm	AAF57940
Drosophila melanogaster	Dm	CAA21317
Drosophila melanogaster	DmLys	CAA80225
Drosophila melanogaster	DmLysB	AAF47448
Drosophila melanogaster	DmLysC	AAF47449
Drosophila melanogaster	DmLysD	AAF47450
Drosophila melanogaster	DmLysE	AAF47451
Drosophila melanogaster	DmLysP	AAF47452
Drosophila melanogaster	DmLysS	AAF47453
Drosophila melanogaster	DmLysX	AAF47445
Heliothis virescens	HcLys	AAD00078
Hyphantria cunea	HcLys	AAA84747
Hyalophora cecropia	HceLys	AAA29189
Manduca sexta	MsLys	AAB31190
Musca domestica	MdLys	PC4062
Rhodnius prolixus	RpLys	ABX11554
Rhodnius prolixus	RpLys1	ABX11553
Triatoma brasiliensis	TbLys	AAU04569
Triatoma infestans	TiLys2	ABI94387
