# Supplementary material for: Expression pattern of glycoside hydrolase genes in Lutzomyia longipalpis reveals key enzymes involved in larval digestion
Source: Front Physiol. 2014 Aug 5;5:276. doi: 10.3389/fphys.2014.00276 (PMC4122206; doi:10.3389/fphys.2014.00276)
Supplement: Supplementary file 5 [file DataSheet5.PDF]

```
                                                                                                  CR1
LlChit3      1 -------------------------------------------------------------------FPGRQYSRLVRDPQARARFVRHVAEFIEKYG   31
AgChit10  2040 DGKYTPDDIDSTLCTHIVYGFAVLDRETLTIKTHDSWADIDNRFYERVVEQKRNGAKVTLALGGWNDSLGDKYSKLVRDAAARARFVKHAVEFIEKYD 2137
DmChit3   1924 QGKFVPEDIDANLCTHLIYGFAVLDSKSLTIKTHDSWTDIDNRFYERVVEYKQRGLRVMLAIGGWNDSLGSKYARLVLNSQSRRRFVASVISFLEQHG 2021
AaChit    1273 DGKYTPDDIDSNLCTHIVYGFAVLDRESLTIKTHDSWADIDNQFYERVVEHKRKGTKVTLALGGWNDSLGDKYSKLVRSSSARRAFVKHAVEFIEKYN 1370
                 CR2 *                                                                            CR3
LlChit3     32 FDGLDLDWEYPVCWQVECDKGFPDEKQGFAELVKELAQEFRPRGWLLSSAVSPSKMVIDKGYDVPTLADYFDWIAVMTYDFHGHWDKQTGHVAPLYYY  129
AgChit10  2138 FDGLDLDWEYPVCWQVDCQKGYPDEKEGFAKLVQELAVAFRPRSWLLSAAVSPSKMVIDAGYDVPVLAEYFDWIAVMTYDFHGNWDKQTGHVAPLYYY 2235
DmChit3   2022 FEGLDLAWEFPVCWQVNCNRGNPTEKDGFVALVKELSEAFKENGLILSAAVSPSKMVIDAGYNVFELSPYFDWVAVMTYDFHGHWDMRTGQIAPLFHR 2119
AaChit    1371 FDGLDLDWEYPVCWQVDCKKGFPDEKEGFAELVKELAIEFRPRKWLLSAAVSPSKMVIDAGYDVPVLAEYFDWIAVMTYDFHGHWDKQTGHVAPLYYY 1468

LlChit3    130 PGDTYDYFNANFSIHYWIEKGAPPSKLVMGLPLYGQSFSLADVSNRGMNEKSYGPGEAGEFTRAGGFLAYYEICQRANSGGWTVVRDSQGRIGPYAYK  227
AgChit10  2236 PGDTYDYFNANFSINYWIEKGAPSRKLVMGMPLYGQSFSLADTKRNGLNEKSYGPGEAGEFTRAGGFLSFYEICDKVNRQGWTVRRDPEGRIGPYAYS 2333
DmChit3   2120 GGDENLYLNGNFSIHYWLERGIPNDKLVMGMPMYGQTFTLADQNRRSLNDKTVGPGKAGTFTRADGFLAYYEICEKVVNDDWKVVRDEEGIFGSYAYS 2217
AaChit    1469 PGDTYDYFNANFSINYWIEKGAPSRKLVMGMPLYGQSFSLADSGKNGLNDKSYGPGEAGQFTRAGGFLAFYEICEKVNQNGWAVYRDSEGRIGPYALR 1566
                                          CR4
LlChit3    228 GSQWVSYDDVSEVRRKAQFIREMKLGGGMIWALDLDDFRGRCGCGKHPLLRTLNQELRGLPGQRATDCT  296
AgChit10  2334 GSQWVSYDDVDEIRRKSQFVKRMNLGGGMVWALDLDDFRGRCGCGTHPLLRTMNLELGRISTQAPENCT 2402
DmChit3   2218 GNQWISYDDVTTIRRKSQFIKSLQLGGGMIWALDLDDFRGLCGCGKHPLLRTLSQELLGIPGQKAKDCI 2286
AaChit    1567 GNQWVSYDDVDEIRRKSQFVKKMNLGGGMIWALDLDDFRGRCGCGRHPLLKTMNQELGRITTKRPENCT 1635
```

Figure S5. Amino acid sequence aligment of selected insect chitinases similar to *L. longipalpis* NSFM-88d12 (named as LlChit3). Conserved residues are with black background and consensus alternatives are shaded. Catalytic residues are marked with asterisks and the conserved regions (CRs) are indicated with dotted boxes. The sequences used in the aligment were retrieved from *Anopheles gambiae* (AgChit10: accession number XP_001238192), *Drosophila melanogaster* (DmChit3: EAA46011) and *Aedes aegypti* (AaChit: AAB81850). All sequences except NSFM-88d12 and AaChit have a putative signal peptide (not shown).
